# Spatially local inhibition and synaptic plasticity together enable dynamic, context-dependent integration of parallel sensory pathways

**DOI:** 10.1016/j.celrep.2026.117306

**Published:** 2026-04-24

**Authors:** Qiang Chen, Fred Rieke

**Affiliations:** 1 Department of Neurobiology and Biophysics, University of Washington, Seattle, WA 98195, USA; 2 Lead contact

## Abstract

Retinal ganglion cells have traditionally been grouped into cells that are sensitive to luminance but not spatial structure and cells with responses that are enhanced by spatial structure. Neither category describes mouse Off-transient alpha cells, which respond strongly to spatially homogeneous inputs and are suppressed by spatial structure. We identified two circuit mechanisms that together can explain this unusual spatial selectivity. First, the inhibition that controls responses of these cells is tuned to finer spatial structure than excitation, causing the balance of excitation and inhibition to depend on spatial scale. Second, the excitatory synapses onto these cells undergo strong synaptic depression, and the modulation of that depression by presynaptic inhibition amplifies responses to the transition from spatially structured to homogeneous inputs. A spatiotemporal computational model incorporating these circuit features quantitatively recapitulates the observed responses. These findings reveal how localized inhibition and short-term plasticity jointly create the distinctive spatial selectivity of Off-transient cells.

## In brief

Q. Chen and F. Rieke show how two common circuit mechanisms—integration of distinct excitatory and inhibitory signals and short-term synaptic plasticity—work in concert to create an unusual selectivity of one retinal ganglion cell type for spatially homogeneous inputs.

## Graphical Abstract

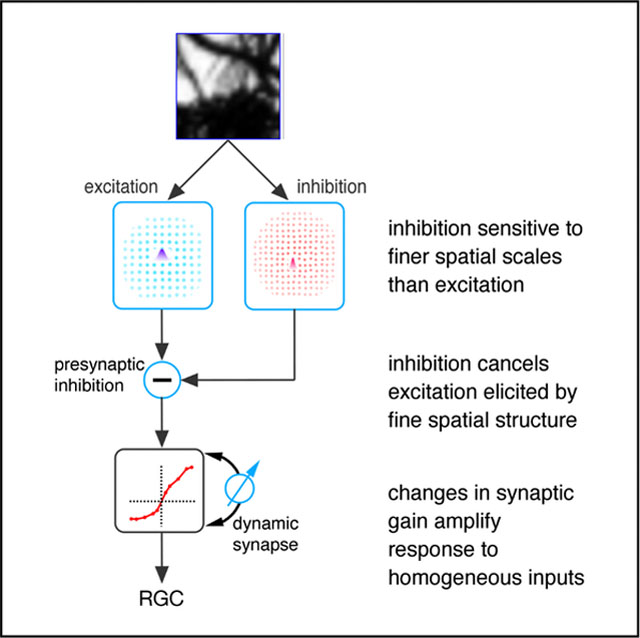

## INTRODUCTION

Interactions between excitation and inhibition (E/I) are a cornerstone of neural computation. Excitatory and inhibitory inputs are often dynamically balanced, and stimulus-dependent modulation of this balance shapes the sensitivity of sensory neurons to specific stimulus features.^1–6^ While many studies focus on changes in E/I balance, less is known about how they interact with other circuit mechanisms to shape circuit computation. Here, we investigate how E/I balance and short-term synaptic plasticity together create an unusual selectivity for spatially homogeneous visual inputs that differentiates one retinal ganglion cell (RGC) type from its counterparts.

Inhibitory circuits shape stimulus selectivity in several ways. Inhibition in many sensory circuits is more broadly tuned (e.g., in visual space or across odors) than excitation.^7,8^ The resulting lateral inhibition sharpens stimulus selectivity by amplifying the differences in responses of neurons that are strongly and weakly activated by a given stimulus. Inhibitory circuits often play a similar sharpening role in the time domain, where temporally delayed inhibition can sharpen temporal tuning by creating a narrow window of opportunity for spiking.^3,9,10^ Excitation and inhibition are often finely balanced, and hence subtle differences in the amplitude or timing of inhibition relative to excitation can have large effects on neural responses.

E/I interactions occur in the context of other circuit mechanisms such as short-term synaptic plasticity.^11,12^ These circuit mechanisms can alter the E/I balance and impact computation. In the hippocampus, for example, excitatory and inhibitory signals exhibit opposite forms of plasticity and the resulting dynamic changes in E/I balance can enhance signals characteristic of place fields.^11,13,14^ In the retina, short-term plasticity at inhibitory synapses has been proposed to selectively suppress responses to repeated stimuli and enhance those to novel stimuli,^15^ and changes in tonic inhibitory transmission can modulate excitatory transmission and increase the sensitivity of retinal outputs in a manner that depends on stimulus history.^16^

Our goal here was to exploit the knowledge of how signals are routed through retinal circuits to study the general issue of how E/I balance works in concert with other circuit mechanisms to control circuit outputs. RGCs typically respond more strongly to spatially structured inputs than to unstructured inputs.^17,18^ Off-transient alpha RGCs (OffT αRGCs) are an exception, responding more strongly to spatially homogeneous inputs.^19,20^ We find that this preference for homogenous inputs emerges from the interaction of two distinct circuit features. First, inhibitory signals are tuned to finer spatial structure than excitatory signals, causing inhibition to preferentially cancel responses to fine spatial structure. This is the opposite of well-studied lateral inhibition.^21,22^ Second, the excitatory synapses onto OffT αRGCs exhibit strong synaptic depression. Presynaptic inhibition recruited by spatially structured inputs relieves this depression, causing strong responses to subsequent spatially homogeneous inputs.

## RESULTS

We start by identifying and characterizing two circuit mechanisms that contribute to the selectivity of OffT αRGCs for spatially homogeneous inputs. First, we show that the inhibitory circuits that control OffT responses are sensitive to smaller spatial scales than the excitatory inputs, causing inhibition and excitation to preferentially cancel for fine spatial scale inputs. This originates from the divergence of common inputs to two parallel pathways that are sensitive to distinct spatial scales. Second, we show that presynaptic inhibition relieves synaptic depression at excitatory synapses and by doing so, enhances responses to spatially homogeneous inputs. Finally, we construct a quantitative model incorporating these two mechanisms and show that it can replicate key features of the OffT αRGC responses.

**Figure 1.**
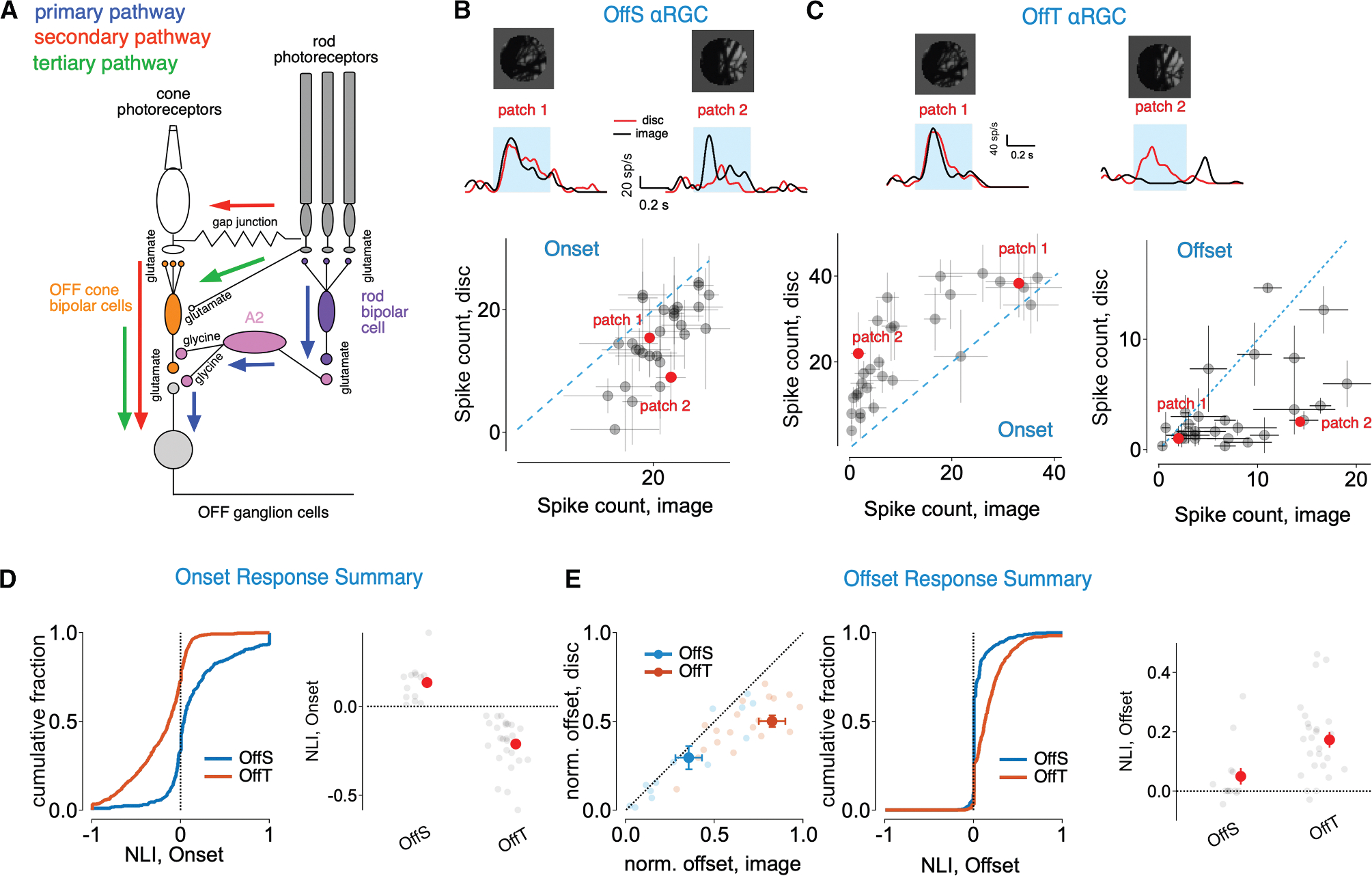
Nonlinear spatial integration across αRGCs (A) Parallel rod signaling pathways. The primary rod pathway (blue arrows) routes rod signals through rod bipolar cells and A2 amacrine cells to ganglion cells. The secondary pathway (red arrows) utilizes rod-cone gap junctions, allowing rod signals to flow through cone bipolar cells to ganglion cells. The tertiary pathway (green arrows) directly connects rods to OFF cone bipolar cells, activating OFF ganglion cells. (B) Spatial integration in Off-sustained (OffS) alpha retinal ganglion cells. (Top) Example spike responses to two image patches and their corresponding equivalent discs. (Bottom) Scatterplot for onset responses, indicating preference for spatially structured stimuli. Each point represents the response to a single image patch and its corresponding linear equivalent disc; error bars represent SEM across 3–6 trials. The brightest pixel in the source image is set to 1; in this recording, that maximum corresponded to 500 R/rod/s*, yielding a mean background of ~100 R/rod/s*. Patch 1 (weighted mean 0.11) and Patch 2 (weighted mean 0.15) are shown as examples; their normalized model differences (0.07 and 0.58, respectively) quantify patch spatial contrast (see STAR Methods) and were randomly sampled from the subset of points lying at approximately the 25th and 75th percentile distances from the unity line in the onset-response comparison. (C) Spatial integration in Off-transient (OffT) alpha retinal ganglion cells. (Top) Example spike responses to two image patches (black traces) and their corresponding linear-equivalent discs (red traces) during stimulus presentation (blue shading). (Bottom) Scatterplots comparing spike counts for image patches versus equivalent discs for onset (left) and offset (right) responses. Error bars represent SEM. Patch 1 and Patch 2 are the same patches shown in (B). (D) Onset response summary. (Left) Cumulative distribution of onset nonlinearity indices across all image-patch/disc pairs for OffS (blue) and OffT (orange) cells. Note that OffT cells predominantly show negative NLIs. (Right) Summary of mean onset NLIs for individual cells (gray circles) and population means ± SEM (red circles) for OffS and OffT types. *n* = 13 cells, OffS αRGC; *n* = 25 cells, OffT αRGC. (E) Offset response summary. (Left) Offset responses normalized to the mean of onset responses, comparing responses to linear-equivalent discs versus natural image patches for all alpha RGC subtypes. Each point plots the mean response for a single cell across all linear equivalent discs (*y* axis) plotted against the mean across all sampled patches (*x* axis). This comparison reveals that OffT cells (orange) exhibit substantially stronger offset responses relative to OffS cells. (Middle) Cumulative distribution of offset nonlinearity indices for OffS and OffT cells. (Right) Summary of mean offset NLIs for individual cells (gray circles), and population means (red circles) for OffS and OffT types. Note: the offset NLI was set to 0 when the offset response was <3 spikes for both disc and image.

Our studies build on prior knowledge of the three parallel pathways that convey rod signals through the rodent retina under these conditions^23–29^ (Figure 1A; Figure S1): (1) the primary rod bipolar → A2 amacrine pathway, (2) the secondary rod-cone gap junction pathway, and (3) the tertiary direct rod-to-off cone bipolar cell pathway. At the mesopic light levels we study here (~100 R*/rod/s), all three pathways are active and converge onto OffT αRGCs; under these conditions, the preference of these cells for homogeneous inputs is strong. We leverage this understanding of rod pathway organization to investigate how interactions between these parallel circuits shape the distinctive spatial selectivity of OffT cells.

### Off-transient αRGCs exhibit robust homogeneity preference at stimulus onset and offset

We assessed how RGCs integrated spatial inputs within their receptive field (RF) center by comparing responses to flashed natural image patches with responses to spatially homogeneous “linear-equivalent” discs^18,30^ (Figures 1B and 1C). For each natural image patch, we computed a corresponding uniform disc whose intensity was given by the weighted linear sum of the patch’s pixel intensities, with weights defined by a Gaussian profile matching the cell’s RF center (see STAR Methods). All stimuli were confined to the RF center estimated for each cell at the start of recording (see STAR Methods). If a RGC integrated spatial inputs linearly, responses to the natural image patch and its corresponding linear-equivalent disc should be near identical. Substantial disparities in these responses thus indicated nonlinear spatial integration (see STAR Methods for controls).

Natural image patches exhibited substantial variability in spatial structure and local contrast (Figures 1B and 1C; Figure S7). For each cell, the patches shown were selected from a large randomly sampled pool of candidate patches drawn from a given natural image and ranked using a model-based measure of spatial structure; a subset of patches spanning the full range from highly structured to nearly uniform was then presented (see STAR Methods). When we compared responses to natural images with those to the corresponding homogeneous discs, we found that Off αRGC subtypes responded to spatially structured inputs very differently (Figures 1B–1E). At stimulus onset, Off-sustained (OffS) αRGCs typically exhibited enhanced responses to spatially structured natural image patches compared with uniform linear-equivalent discs (Figure 1B). OffS cells responded predominantly at stimulus onset and generated little or no separable response at stimulus offset (Figures 1B and 1E). OffT αRGCs, in the vast majority of cases, showed stronger responses to linear-equivalent discs at stimulus onset, indicating a preference for spatial homogeneity (Figure 1C, left). OffT, but not OffS, αRGCs also exhibited robust responses to the transition from a spatially structured image to a uniform background (i.e., at stimulus offset; Figure 1C, right), highlighting their sensitivity to—and preference for—the return to spatial homogeneity. Consistent with this interpretation, OffT offset responses were much stronger for image patches than corresponding linear-equivalent discs.

We quantified the deviation from linear spatial integration for each image patch using a nonlinearity index (NLI), defined as follows:

$$NLI=\frac{r_{\mathrm{image}}\;-\;r_{\mathrm{disc}}}{r_{\mathrm{image}}\;+\;r_{\mathrm{disc}}}.$$

Here, $r_{\mathrm{image}}$ and $r_{\mathrm{disc}}$ are the average spike counts in response to the original natural image patch and the corresponding linear-equivalent disc. The NLI compares the measured responses to a structured patch with that to the linear-equivalent disc. An NLI of 0 indicates linear spatial integration, whereas nonzero values indicate a sensitivity to spatial structure not predicted by linear integration.

We used the same NLI definition for responses at stimulus onset and offset. The interpretation differs, however, because the stimulus conditions differ (i.e., background→stimulus versus stimulus→background). At stimulus onset, positive NLIs indicate a preference for spatial structure (stronger responses to the onset of the image than the disc), whereas negative NLIs indicate a preference for spatial homogeneity (stronger responses to the onset of the disc than the image). At stimulus offset, the NLI quantifies responses elicited by the return to the uniform background: positive values indicate homogeneity preference—cells respond more strongly to the offset of a spatially structured image and the return to the background than they do to the offset of the uniform disc and return to the background. We computed an NLI for each image patch and summarized each cell’s homogeneity preference by averaging NLIs across patches (cell-level NLI). Using this metric, NLIs for OffT cells were significantly negative at onset and significantly positive at offset relative to OffS αRGCs (and other αRGCs subtypes; Figures 1D and 1E). This analysis indicates a preference for the onset of homogeneous stimuli (i.e., linear-equivalent discs) and offset of spatially structured stimuli and return to homogeneity (i.e., the offset of image patches).

Together, the findings summarized in Figure 1 demonstrate that OffT αRGCs exhibit a robust homogeneity preference. These distinctive responses of OffT αRGCs to the onset and offset of spatially structured stimuli differentiate them from other αRGC subtypes.

### Synaptic mechanisms of OffT αRGC homogeneity preference

Having established the distinctive homogeneity preference of OffT αRGCs, we next investigated the underlying synaptic mechanisms mediating these responses using naturalistic stimuli and controlled artificial stimuli.

**Figure 2.**
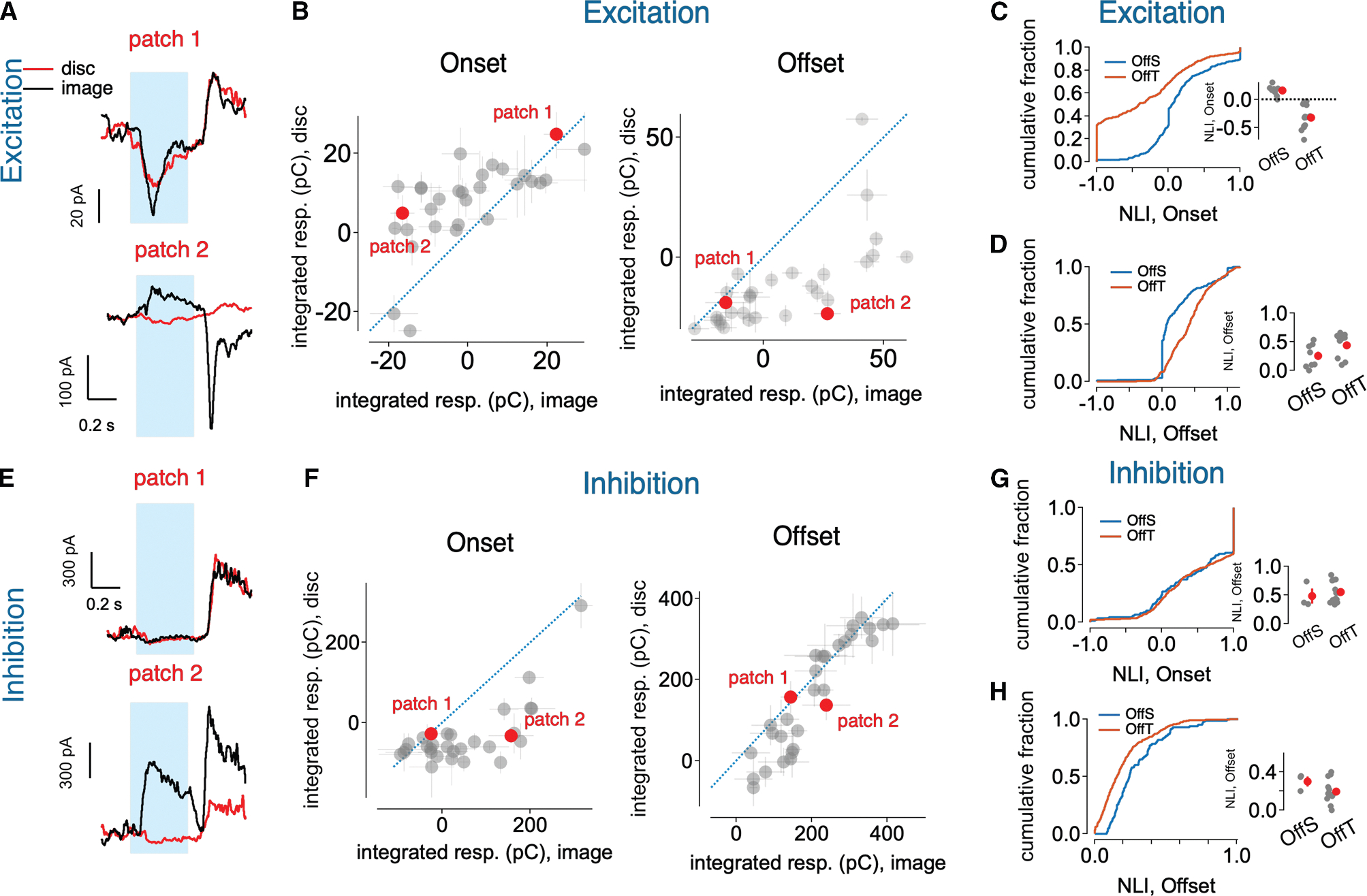
Synaptic mechanisms underlying OffT αRGC responses to natural images (A) Example excitatory current traces recorded from an OffT αRGC in response to two natural image patches (black) and their corresponding linear-equivalent discs (red). The blue shaded region indicates the stimulus presentation period. Patch 1 and Patch 2 are the same patches shown in Figures 1B and 1C. (B) Scatterplots comparing excitatory input charge transfer (pC) in response to natural image patches versus linear-equivalent discs. (Left) At stimulus onset, excitatory inputs show stronger responses to linear-equivalent discs than to natural image patches (points above the unity line). (Right) At stimulus offset, excitatory inputs show robust responses to the transition from natural images to a uniform background (points below the unity line). (C) Cumulative distributions of onset nonlinearity indices (NLIs) for excitatory inputs across different αRGC types. (Left) Cumulative distributions for OffS (blue) and OffT (orange) cells. (Right) Summary of mean excitatory onset NLIs (averaged across all patches for a given cell) for individual cells (gray circles) and population means ± SEM (red circles). *n* = 8 cells, OffS; *n* = 9 cells, OffT αRGC. (D) Same as (C) but for offset responses. (E) Example inhibitory current traces recorded from an OffT αRGC in response to two natural image patches (black) and their corresponding linear-equivalent discs (red). Patch 1 and Patch 2 are the same patches shown in (A and B). (F) Scatterplots comparing inhibitory input (left: onset; right: offset) charge transfer (pC) in response to natural image patches versus linear-equivalent discs. (G) Cumulative distributions of onset nonlinearity indices for inhibitory inputs across different αRGC types. (Left) Cumulative distributions for OffS (blue) and OffT (orange) cells. (Right) Summary of mean inhibitory onset NLIs for individual cells (gray circles) and population means ± SEM (red circles). *n* = 3 cells, OffS αRGC; *n* = 10 cells, OffT αRGC. (H) Same as (G) but for offset responses.

We recorded excitatory and inhibitory synaptic inputs to OffT αRGCs in response to natural image patches and their linear-equivalent discs (Figure 2). At stimulus onset, excitatory inputs to OffT αRGCs demonstrated a clear preference for homogeneous discs, evident from the preponderance of data points that fall above the unity line and the negative onset NLIs (Figures 2A–2C). Spatial structure often elicited a reduction in tonic or baseline excitatory current compared with homogeneous discs (e.g., patch 2 in Figure 2A)—suggestive of presynaptic inhibition that was preferentially recruited by spatially structured stimuli. Conversely, inhibitory synaptic inputs displayed stronger responses to structured natural images compared with linear-equivalent discs, indicated by data points falling below the unity line and positive onset NLIs (Figures 2E–2G). These findings suggest that spatial structure in natural images robustly activates inhibitory pathways, likely involving both direct inhibition to OffT αRGCs and presynaptic inhibition to the bipolar cells that provide excitatory input to OffT cells.

The return from a structured image to spatial homogeneity at stimulus offset often substantially increased excitatory synaptic inputs to OffT αRGCs (Figures 2B–2D), consistent with the positive offset NLI previously observed in the spike response. Patches that elicited a robust increase in excitatory synaptic input at stimulus offset also showed a clear suppression of excitatory input at stimulus onset (e.g., patch 2 in Figure 2).

**Figure 3.**
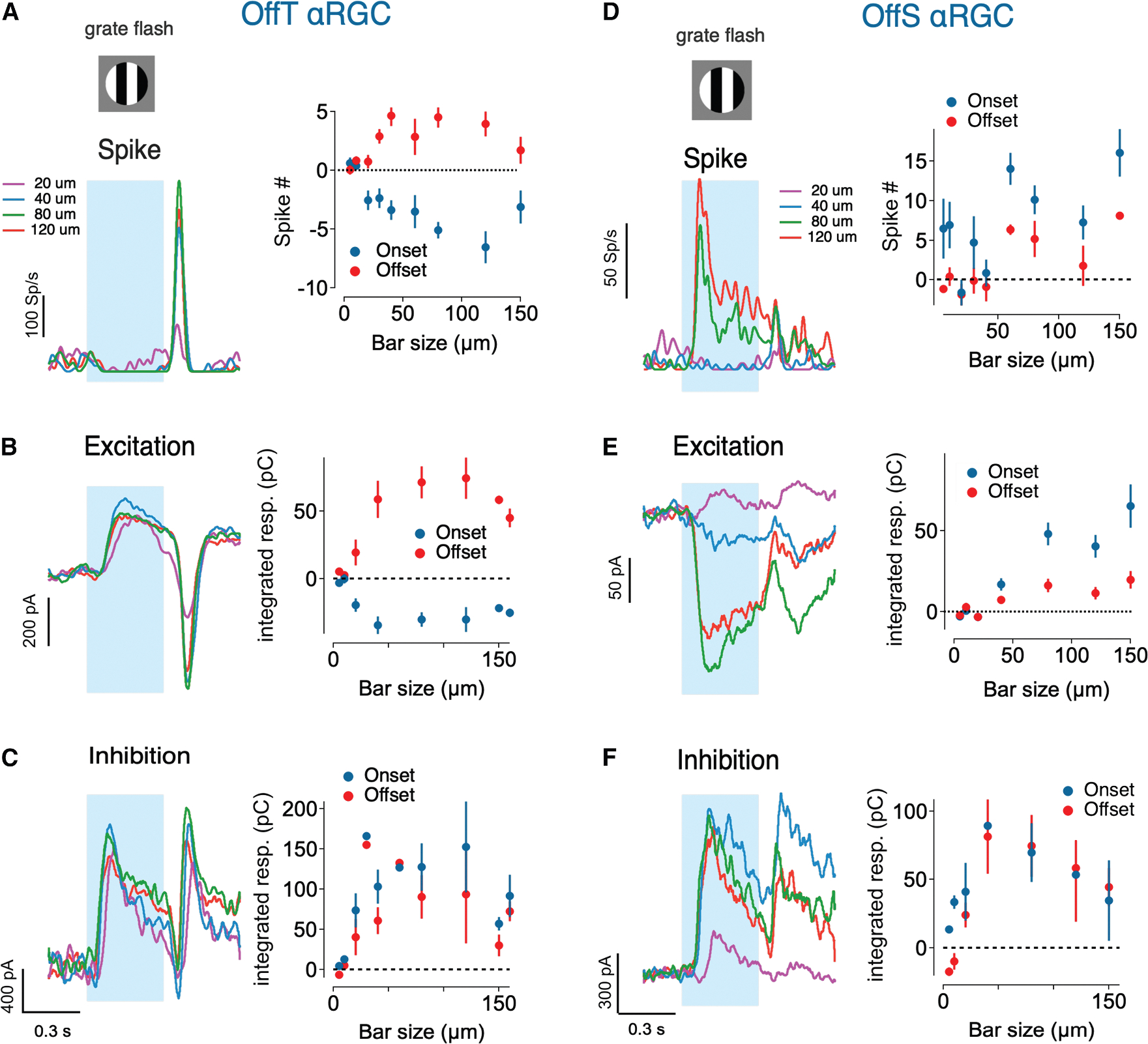
Comparison of synaptic responses to flashed gratings between OffT and OffS αRGCs (A) Response properties of OffT alpha retinal ganglion cells to flashed gratings. (Left) Example spike traces for four different bar sizes with blue shading indicating the flash duration. (Right) Quantified spike responses as a function of bar size for both onset (blue circles) and offset (red circles) of the flash. Note that negative spike responses at onset indicate suppression of the spike rate relative to baseline. *n* = 30 cells. (B) Excitatory synaptic input recordings from OffT αRGCs in response to flashed gratings. (Left) Example excitatory current traces for the four bar sizes. (Right) The integrated excitatory response as a function of bar size at both onset (blue) and offset (red). Excitatory responses exhibit suppression during grating presentation and enhancement at stimulus offset. *n* = 16 cells. (C) Inhibitory synaptic input recordings from OffT αRGCs in response to flashed gratings. (Left) Example inhibitory current traces for the four bar sizes. (Right) The integrated inhibitory response as a function of bar size at both onset (blue) and offset (red). *n* = 8 cells. (D–F) same as (A–C), but for OffS αRGCs. *n* = 13, 9, and 7 cells for spike, excitatory, and inhibitory recordings, respectively.

To more precisely isolate the impact of spatial structure, we conducted additional experiments using flashed gratings with a range of bar sizes (Figure 3). As in the other experiments, stimuli were positioned relative to each cell’s RF center estimated at the start of the recording (see STAR Methods). For gratings specifically, we set the grating phase so that a luminance zero-crossing (the boundary between bright and dark bars) fell at the RF center, minimizing any net luminance change within the RF center at stimulus onset and thereby emphasizing responses driven by spatial structure rather than changes in mean intensity. The onset of a flashed grating strongly suppressed OffT αRGC spike responses through a pronounced suppression of baseline excitatory input and an increase in inhibitory input (Figures 3A–3C). At grating offset, OffT αRGCs exhibited robust spike responses characterized by a marked increase in excitatory input (Figures 3A and 3B). These temporal response profiles differed from those of OffS αRGCs, for which excitatory and inhibitory synaptic inputs increased at both grating onset and offset (Figures 3D–3F). Unlike the OffT responses, the OffS spiking response to the offset of a grating was generally weak (Figures 3D–3F). This distinction emphasizes an unexpected difference in the synaptic mechanisms controlling responses of these two Off αRGC types.

Because αRGC response properties have been reported to vary with retinal location,^31,32^ we compared the homogeneity preference of OffT αRGCs from dorsal versus ventral retina. OffT αRGCs from both regions exhibited comparable preferences for spatially homogeneous stimuli and similar patterns of excitatory and inhibitory synaptic input (Figure S2). Consistent with this, all OffT αRGCs in our dataset showed a negative NLI at the onset of a flashed natural image and a positive NLI at image offset, indicating a preference for spatial homogeneity and for the return to homogeneity. These results indicate that the homogeneity preference reported here does not depend strongly on dorsal versus ventral retinal location.

Several aspects of the experiments illustrated in Figures 2 and 3 suggest potential circuit mechanisms that could generate the OffT αRGCs’ unusual homogeneity preference. First, spatially structured stimuli appear to recruit inhibition both directly on OffT αRGCs and presynaptically on their bipolar inputs, as evidenced by the suppression of baseline excitation. Second, the pronounced response at the offset of spatial structure hints at a gain control mechanism. The experiments below probe each of these mechanisms in more detail.

### Spatially localized inhibition creates scale-dependent E/I balance in OffT αRGCs

We next studied interactions between E/I. We start by examining their spatial-temporal properties in more detail. At the mesopic light levels we studied, E/I are routed through parallel pathways, with inhibitory synaptic inputs to Off αRGCs derived predominantly through the primary rod pathway and excitatory inputs derived from a mixture of primary, secondary, and tertiary rod pathways (Figure 1A).^23,25^ This parallel routing suggested that the properties of the nonlinear RF subunits that control responses to spatial structure might differ for excitatory and inhibitory synaptic inputs to Off αRGCs.

**Figure 4.**
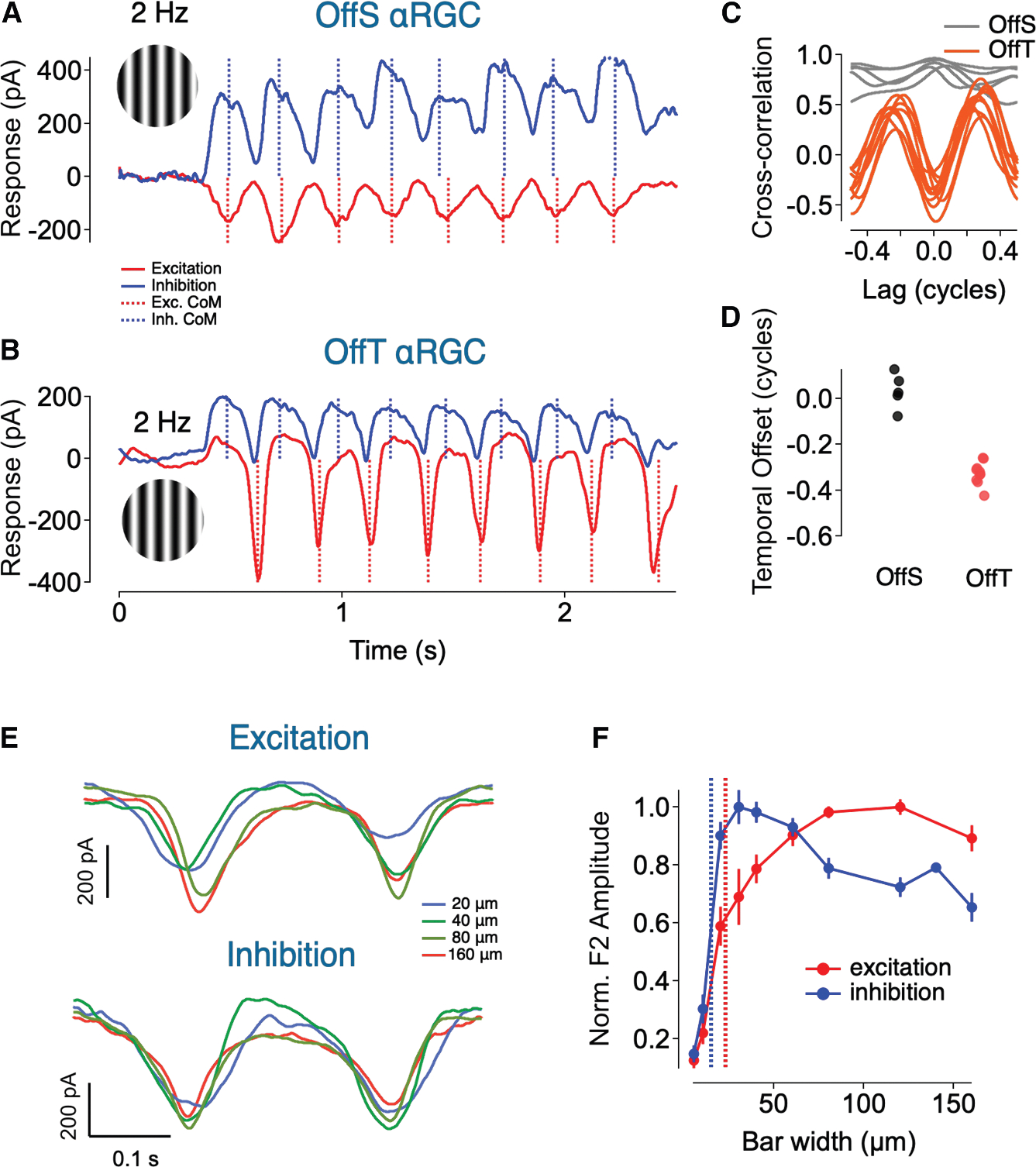
Subunits in synaptic inputs of αRGCs (A and B) Overlay of excitatory and inhibitory currents recorded from OffS (A) and OffT (B) αRGCs at bar size of 40 μm. Dotted lines indicate centers of mass (CoMs) for each cycle. (C) Cross-correlation between excitatory and inhibitory inputs for OffS and OffT αRGCs. Lag time is normalized to the F2 response cycle of 500 ms. *n* = 5 cells, OffS αRGC; *n* = 10 cells, OffT αRGC. (D) Temporal offset between excitatory and inhibitory inputs for OffS and OffT αRGCs, calculated using the CoM of the responses. Temporal offset time is normalized to the F2 response cycle of 500 ms. (E) Example excitatory (upper) and inhibitory (lower) currents recorded in response to contrast-reversing gratings of different bar widths. (F) Normalized F2 amplitude of excitatory and inhibitory inputs as a function of bar width. Vertical dashed lines mark the normalized bar width that elicited the half-maximal F2 response; we define this value as the subunit radius. *n* = 13 cells for excitation, *n* = 20 cells for inhibition.

We characterized the subunit properties of excitatory and inhibitory inputs using whole-cell voltage-clamp recordings, while employing a classic test for nonlinear subunits using contrast-reversing gratings.^33,34^ In the case of linear spatial integration, responses to the bright and dark bars of a grating will cancel, resulting in no response from the RGC (Figure S3B). However, this cancellation does not occur with nonlinear subunits, and the RGC generates distinctive responses at twice the grating modulation frequency (“F2” responses, Figures 4A and 4B; Figure S3B).

Contrast-reversing gratings elicited excitatory and inhibitory inputs to OffS and OffT cells with distinct temporal relationships. In OffS αRGCs, excitatory and inhibitory synaptic inputs increased and decreased together with minimal temporal offset. In OffT αRGCs, inhibitory input preceded excitatory input by nearly half a grating cycle (Figures 4A–4D). These distinct temporal offsets cannot be easily explained by misalignment of the grating and the RF (see STAR Methods) and instead reflect differences in the properties of the circuits controlling synaptic inputs to OffT and OffS cells.

This temporal offset is consistent with a circuit in which A2 amacrine cells provide both direct inhibition to the ganglion cell and presynaptic inhibition to Off bipolar terminals. For OffT cells, presynaptic inhibition appears to suppress the expected increase in excitatory synaptic input, which instead occurs only when presynaptic inhibition subsides. Excitatory input in these cells is further amplified by synaptic plasticity, an effect that we explore in the second half of the paper.

Subunit sizes, inferred from the dependence of the response on grating bar width (Figures S3C–S3F), were substantially smaller than the overall RF center size (Figure S3A). Notably, excitatory and inhibitory inputs showed distinct spatial tuning: inhibitory inputs peaked at smaller bar widths than excitatory inputs and as the bar width increased, inhibitory inputs declined, while excitatory inputs remained relatively constant (Figure 4F).

To understand the suppression of inhibition at larger spatial scales, we constructed a spatial center-surround model of inhibitory subunits (see STAR Methods; Figure S4). Each subunit was modeled as a difference-of-Gaussians RF, embodying center-surround antagonism. By adjusting the surround strength and extent, the model accurately replicated the observed decrease in inhibitory F2 responses for wider bars (Figures S4C–S4E). This strong, local surround suppression narrows inhibitory spatial integration and differentiates it from the broader integration of excitatory inputs.

The different spatial profiles of inhibitory and excitatory subunits create a spatially dependent ratio of inhibition to excitation. Fine spatial structure drives strong local inhibition, suppressing excitation and producing a high inhibition-to-excitation ratio. But for larger, uniform stimuli, inhibitory subunits experience surround suppression and thereby contribute less inhibition, allowing excitation to dominate.

### Spatially local inhibition arises from inter-pathway glycinergic modulation by A2 amacrine cells

Figures 1 and 2 show that OffT αRGCs robustly prefer spatially homogeneous stimuli, consistent with presynaptic inhibition suppressing excitatory responses to fine spatial structure. To identify the circuit elements mediating this inhibition, we focused on interactions between the rod pathways that converge onto Off cone bipolar cells. The primary rod pathway provides glycinergic inhibitory input to Off cone bipolar cells through A2 amacrine cells. Direct inhibitory synaptic input to OffT and OffS αRGCs is also largely glycinergic^19,35^ and persists in NBQX, which should abolish light-evoked responses in essentially all amacrine cells except A2 amacrines.^19,35,36^ Hence, A2 amacrine cells shape Off αRGC responses through both pre and post-synaptic inhibition. To test whether this pathway is necessary for homogeneity preference, we blocked it using two complementary approaches.

**Figure 5.**
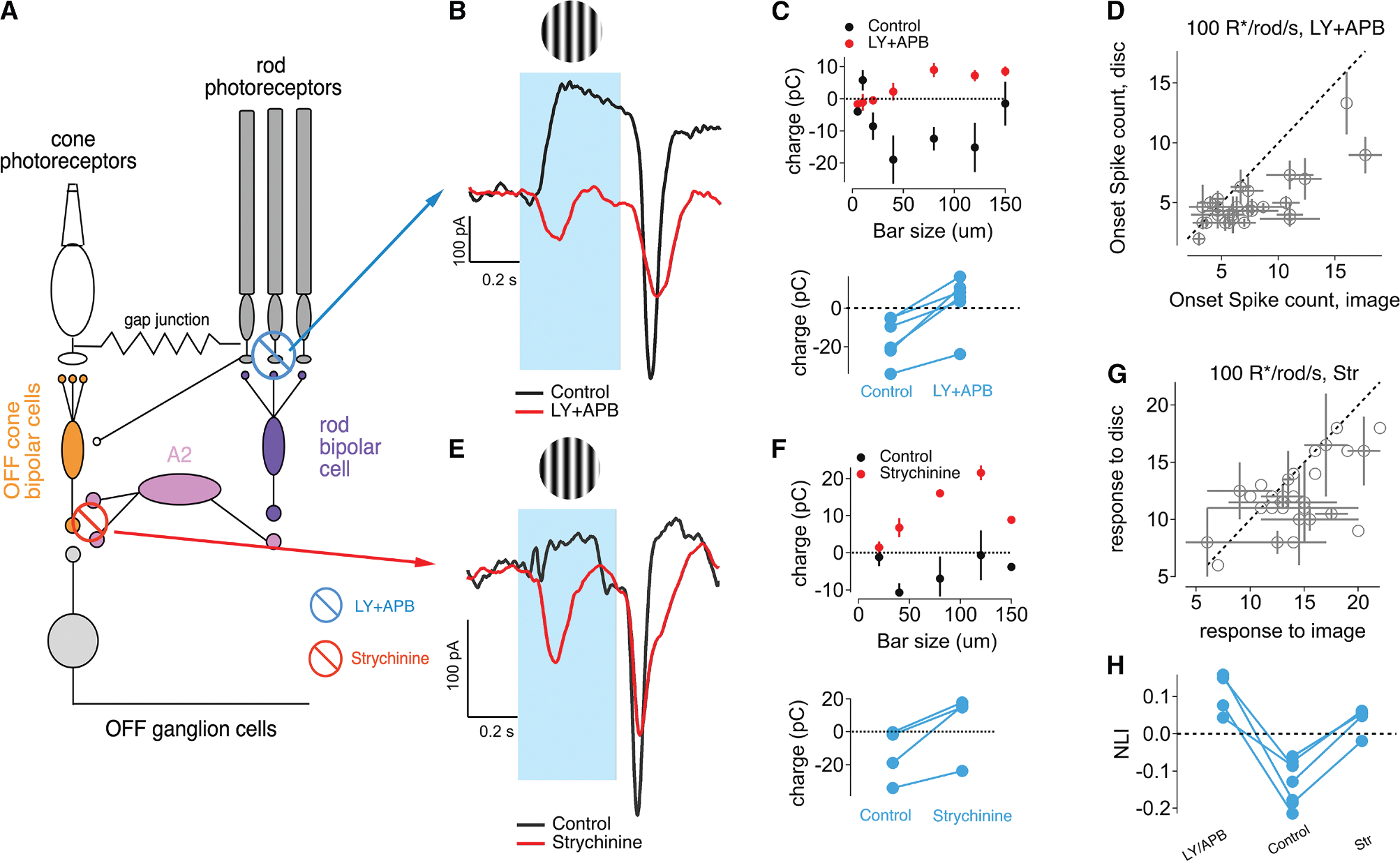
Pharmacological profiles of presynaptic inhibition in OffT αRGCs (A) Schematic showing parallel rod pathways in the retina with sites of pharmacological intervention. LY341495+APB (blue) blocks the rod→rod bipolar cell transmission, while strychnine (red) blocks glycinergic transmission from A2 amacrine cells. (B) Representative excitatory current traces in response to flashed gratings under control conditions (black) and with LY341495 (10 μM) +APB (10 μM) application (red) to block the primary rod pathway. Blue shading indicates grating presentation. (C) (Top) Quantification of excitatory charge transfer in response to onset of gratings with different bar sizes under control (black) and LY341495+APB (red) conditions. (Bottom) Paired comparison of excitatory charge transfer for individual cells under control and LY341495+APB conditions. *n* = 4 cells. (D) Comparison of OffT αRGC onset spike responses to natural image patches (*x* axis) versus linear-equivalent discs (*y* axis) under LY341495+APB treatment at 100 R*/rod/s. (E) Representative excitatory current traces in response to flashed gratings under control conditions (black) and with strychnine (0.5 μM) application (red) to block glycinergic transmission. (F) (Top) Quantification of excitatory charge transfer in response to gratings with different bar sizes under control (black) and strychnine (red) conditions. (Bottom) Paired comparison of excitatory charge transfer for individual cells under control and strychnine conditions. *n* = 4 cells. (G) Comparison of OffT αRGC onset spike responses to natural image patches versus linear-equivalent discs under strychnine treatment at 100 R*/rod/s. (H) Summary of nonlinearity indices (NLIs) for onset spike responses under control conditions, LY341495+APB treatment, and strychnine treatment at 100 R*/rod/s. Each line represents an individual cell. *n* = 4 cells (LY + APB); *n* = 4 cells (strychnine).

First, we disrupted the rod-to-rod bipolar synapse using a cocktail of agonists and antagonists (APB and LY341495) of the mGluR6 glutamate receptors expressed by On bipolar cells (Figures 5A–5C).^23,24,37^ LY341495 can also antagonize mGluR5, which is expressed at On-bipolar dendrites and has recently been shown to modulate photoreceptor output via retrograde signaling.^38^ We used a mixture of APB and LY341495 that suppressed On-pathway light-evoked responses while preserving tonic excitatory drive and thus avoided aberrant signaling associated with large changes in tonic On pathway activity. Suppressing light-dependent rod bipolar input to A2 amacrine cells in this way revealed robust excitatory responses to the onset of spatially structured stimuli—responses that are normally absent (Figures 5A–5C). Similarly, application of strychnine to block glycinergic transmission—the inhibitory transmitter released by A2 cells^39^—revealed strong onset excitation to spatial structure (Figures 5E and 5F). Both manipulations also attenuated the robust offset responses normally observed, supporting the role of presynaptic inhibition in regulating synaptic gain across stimulus transitions.

When tested with natural images, both pharmacological interventions abolished the homogeneity preference, inverting the normal response pattern such that OffT αRGCs now preferred spatially structured images over linear-equivalent discs (Figures 5D, 5G, and 5H). This confirms that the homogeneity preference requires A2-mediated inhibition rather than being an intrinsic property of the excitatory pathway.

The effects of On-pathway blockade are closely related to earlier observations using polarity-reversing checkerboard stimuli in OffT αRGCs (PV5 cells), where checkerboard reversals evoked little spiking under control conditions and blocking On-pathway signaling with APB revealed robust onset responses.^19^

Collectively, these findings support a model in which the homogeneity preference in OffT αRGCs arises from the interaction between parallel pathways, rather than from mechanisms confined to the primary rod pathway. The key player in this interaction appears to be A2 amacrine cells, which provide both presynaptic inhibition to Off cone bipolar cells and direct inhibition to OffT αRGCs. This dual inhibitory action effectively suppresses excitatory signals from secondary and tertiary pathways in response to spatial structure, thereby shaping the unique response properties of OffT αRGCs to natural images.

### Light-level-dependent shifts in OffT αRGC spatial computation

**Figure 6.**
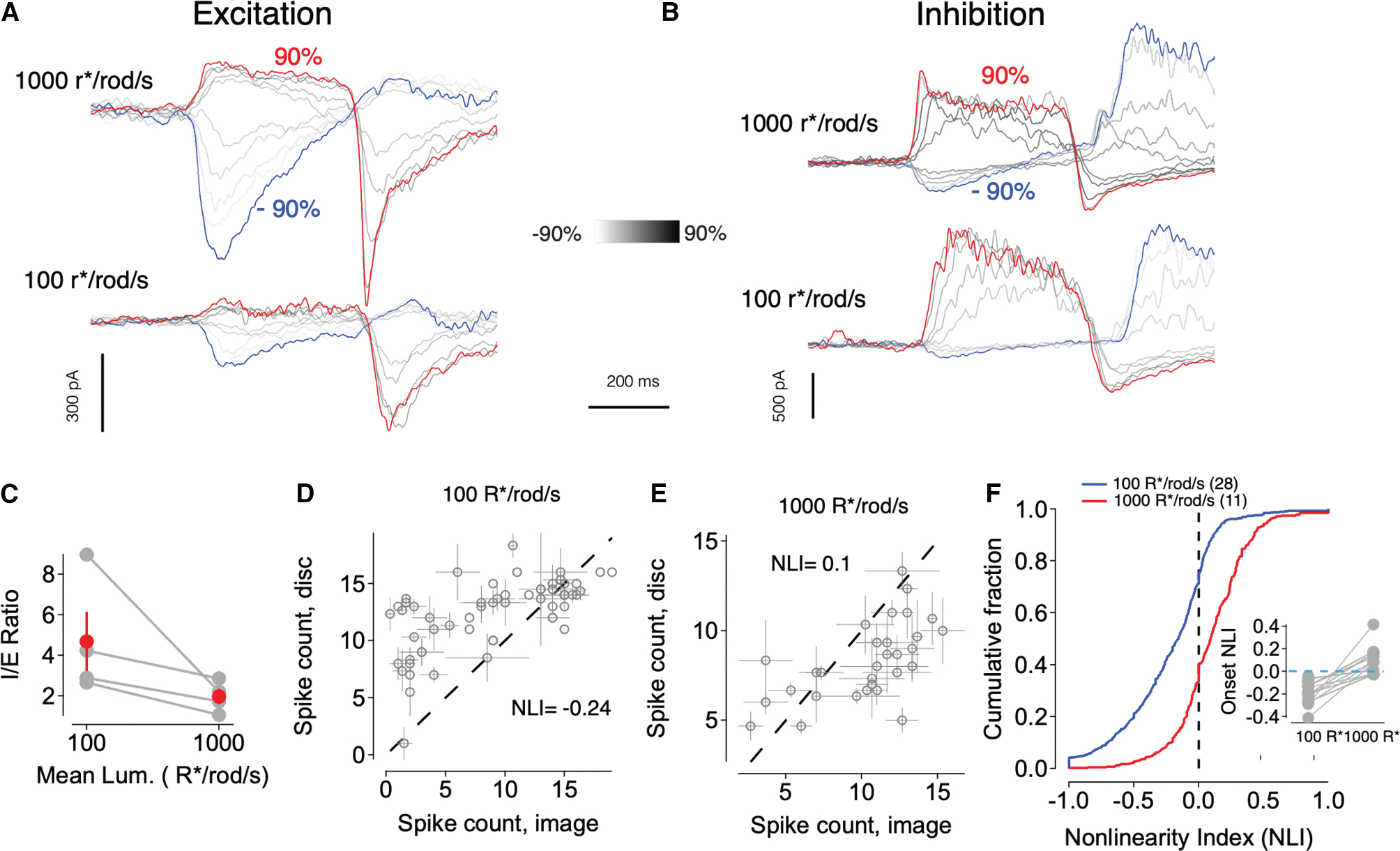
Luminance-dependent spatial encoding in OffT αRGCs (A) Excitatory currents recorded from an example OffT αRGC in response to spot stimuli at a fixed mean light level across contrast steps. Red traces highlight +90% contrast steps, and blue traces highlight −90% contrast steps. (B) Inhibitory currents recorded from the same cell under the same conditions as in (A). Red traces highlight +90% contrast steps, and blue traces highlight −90% contrast steps. (C) I/E ratio for individual cells (gray), and population average (red) at the two light levels. (D) Spike count responses of an example OffT αRGC to natural image patches versus linear-equivalent discs at 100 R*/rod/s. Each point represents one image patch. (E) Responses of the same cell at 1,000 R*/rod/s. (F) Cumulative distributions of nonlinearity index (NLI) values across image/disc pairs and population of OffT αRGCs at 100 R*/rod/s (blue, *n* = 28 cells) and 1,000 R*/rod/s (red, *n* = 11 cells).

Given that A2-mediated inhibition via the primary rod pathway is required to generate the homogeneity preference in OffT αRGCs, we next asked how this computation changes with light adaptation. As ambient light levels increase, excitatory inputs to cone bipolar cell dendrites increase in strength, the primary rod pathway becomes less dominant, and A2 amacrine cells are driven predominantly through electrical coupling with On cone bipolar cells.^23,25^ This shift in input source might alter the strength and spatial organization of A2-mediated inhibition. To examine the functional consequences of this circuit reconfiguration, we first assessed whether the balance of excitatory and inhibitory synaptic inputs to OffT αRGCs changes with light level. At 1,000 R*/rod/s—now within the photopic range—compared with 100 R*/rod/s in the mesopic range, we observed a clear reduction in inhibitory conductances relative to excitatory conductances (Figures 6A–6C). This shift in E/I balance produced a reversal of spatial sensitivity: after adaptation to photopic conditions, OffT αRGCs responded more strongly to spatially structured image patches than to homogeneous discs—opposite the pattern observed under mesopic conditions (Figures 6D–6F).

These results demonstrate that light-level-dependent shifts in rod pathway routing alter both the strength of A2-mediated inhibition and the spatial computations performed by OffT αRGCs, highlighting the essential role of A2-mediated pathway in dynamically shaping spatial computations in the retina.

### Short-term plasticity and glycinergic inhibition underlie history-dependent excitation in OffT αRGCs

We next investigated the robust rebound of excitatory input observed at the offset of spatially structured stimuli (e.g., Figures 2A and 3B). We hypothesized that this rebound reflects a history-dependent modulation of synaptic gain, mediated by presynaptic glycinergic inhibition and short-term plasticity at the bipolar terminal. This mechanism provides a potential circuit-level explanation for the functional divergence between transient and sustained Off αRGC pathways, in which similar spatial inputs give rise to qualitatively different temporal and polarity-specific responses. This idea is consistent with previous work,^25^ which showed that brief periods of reduced drive potentiate subsequent responses via recovery from vesicle depletion or disinhibition at bipolar outputs.

**Figure 7.**
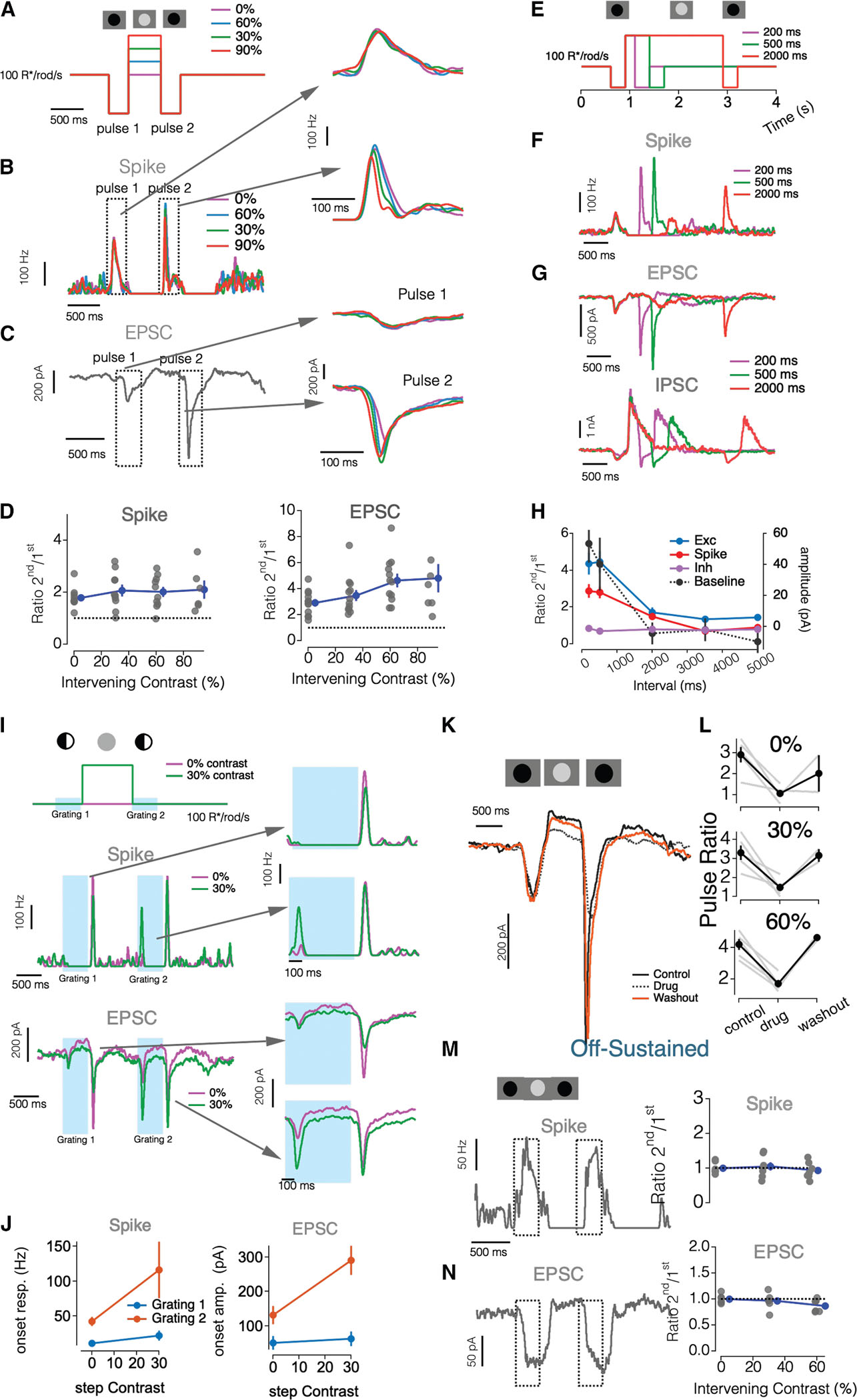
Temporal dynamics of synaptic interactions in Off αRGCs (A–H) Bright interstimulus intervals enhance subsequent light responses in OffT αRGCs. (A) Stimulus protocol showing two negative light pulses (300 ms, −90% contrast) separated by various intervening contrasts (0%–90% contrast, 500 ms). (B) Spike responses of an OffT RGC to paired pulses. Insets: expanded views of responses to pulse 1 (top) and pulse 2 (bottom), aligned to pulse onset. (C) Excitatory postsynaptic currents (EPSCs) evoked by paired pulses in the same cell as (B). (D) Spike (left) and EPSC (right) paired-pulse ratio (pulse 2/pulse 1 peak amplitude) as a function of interpulse contrast. Error bars, ±SEM across cells. (E) Stimulus protocol showing two negative light pulses (300 ms, −90% contrast) separated by variable duration intervals (200 ms, 500 ms, and 2,000 ms; 60% contrast). (F) Spike responses of an OffT αRGC to paired pulses separated by different intervals. Traces show responses for 200-ms (magenta), 500-ms (green), and 2,000-ms (red) intervals. *n* = 12 cells. (G) Synaptic currents (excitatory, EPSC; inhibitory postsynaptic current, IPSC) evoked by paired pulses in the same cell as (F). *n* = 7 cells. (H) Spike (blue), EPSC (red), IPSC (pink), and baseline (black) paired-pulse ratios as a function of interpulse interval duration. Error bars, ±SEM across cells. *n* = 5 cells. (I) (Upper) Stimulus protocol showing two sequential grating stimuli (500 ms, 90% spatial contrast) separated by various intervening contrast steps (0%–30% contrast, 1,000 ms). (Middle) Spike responses to two sequential grating stimuli (grating 1 and grating 2, 500 ms each). Insets: expanded views of responses to grating 1 and grating 2. *n* = 4 cells. (Lower) EPSCs evoked by sequential grating stimuli in the same cell as (F). *n* = 4 cells. (J) Spike (H) and EPSC (I) response amplitudes to grating 1 and grating 2 as a function of inter-grating contrast (0%–30%). Error bars, ±SEM across cells. (K and L) Glycinergic inhibition is necessary for relief from suppression in OffT αRGCs. (K) Example excitatory current traces in response to paired pulses under control conditions (solid black line), with strychnine application (0.5 μM, dotted black line), and after washout (orange line). (L) Summary of paired-pulse ratios under control, strychnine, and washout conditions for different intervening contrasts (0%, 30%, and 60%). Gray lines show individual cells, black lines with error bars show mean ± SEM. *n* = 5 cells. (M and N) OffS αRGCs show minimal relief from suppression effects. (M) Example spike (left) responses of an OffS αRGC to paired-pulse stimuli with different intervening contrasts (0%, 30%, and 60%). (Right) Paired-pulse ratios for spike responses in OffS αRGCs as a function of intervening contrast. Gray dots show individual cells, and blue line with error bars show mean ± SEM. *n* = 7 cells. (N) Same as (M) but for EPSC in OffS αRGC.

To test this hypothesis, we adapted a paired-pulse paradigm in which two identical dark flashes (−90% contrast, 0.3s) were separated by an intervening contrast step presented on a 100 R*/rod/s background. The contrast of the intervening step ranged from 0 (return to baseline) to positive values (opposing), and we measured both synaptic input and spiking output of OffT αRGCs across conditions (Figures 7A–7D). For both zero-contrast and positive-contrast intervals, the response to the second pulse was substantially larger than that to the first pulse, often as much as 3-fold. During the period between the pulses both firing rate and excitatory synaptic input were substantially reduced. This suggests that brief periods of reduced drive potentiate subsequent responses, likely through recovery from synaptic depression. The strength of this facilitation depended on the duration of the recovery interval, with the paired-pulse ratio decreasing progressively with longer intervals (200–5,000ms) before plateauing (Figures 7E–7H). We also tracked baseline activity (measured immediately before each second stimulus pulse) to monitor the dynamics of tonic release. The baseline gradually returned to pre-stimulus levels over a similar timescale (Figure 7H).

Although our interpretation above focuses on excitatory synaptic dynamics, short-term plasticity can also arise within inhibitory circuits, including amacrine mechanisms mediated by Ca^2+^-permeable AMPARs.^40^ Therefore, we applied the same paired-pulse paradigm while using voltage-clamp recordings to isolate inhibitory synaptic inputs. Unlike excitatory synaptic inputs, inhibitory inputs showed no systematic paired-pulse facilitation or depression and no history-dependent change in inhibitory charge transfer across recovery intervals (Figures 7G and 7H), indicating that the temporal facilitation revealed by this paradigm reflects plasticity in the excitatory pathway rather than short-term dynamics of inhibitory synaptic input under these conditions.

We next asked whether the same mechanism shapes responses to spatially structured stimuli by presenting two successive high-contrast gratings (Figures 7I and 7J). Again, a brighter interval separating the flashed gratings elicited a larger response to onset of the second grating in both spike and excitatory postsynaptic current measurements (Figures 7I and 7J). To determine whether glycinergic inhibition is necessary for this facilitation, we repeated the paired-pulse experiments in the presence of strychnine (Figure 7K and 7L). Suppressing glycinergic transmission eliminated the second-pulse enhancement, confirming that presynaptic inhibition—most likely via A2 amacrine cells—leads to response facilitation.

Finally, we asked whether OffS αRGCs show a similar history-dependent increase in excitatory gain (Figures 7M and 7N). Even with high intervening brightness, OffS cells exhibited little or no facilitation. This lack of facilitation occurs even though the firing rate and tonic excitatory input between the two pulses were strongly suppressed. These results underscore that the interaction between glycinergic inhibition and short-term synaptic plasticity is a distinctive feature of OffT αRGCs and is largely absent from OffS αRGCs. This pathway-specific plasticity provides a mechanistic basis for the functional asymmetry observed throughout the study, including the selective preference of OffT αRGCs for spatial homogeneity and their characteristic response suppression at the onset of spatial structure and rebound at its offset.

Taken together, these experiments identify a dual role for inhibition in shaping OffT αRGC activity. First, it directly suppresses excitatory drive during structured stimuli. Second, an increase in presynaptic inhibition allows bipolar terminals to recover from depression and hence to produce a powerful rebound of excitatory output once inhibition lifts. This mechanism endows OffT, but not OffS, αRGCs with flexible, context-dependent control over their excitatory gain.

### A unified spatial-temporal model reproduces OffT αRGC homogeneity preference

**Figure 8.**
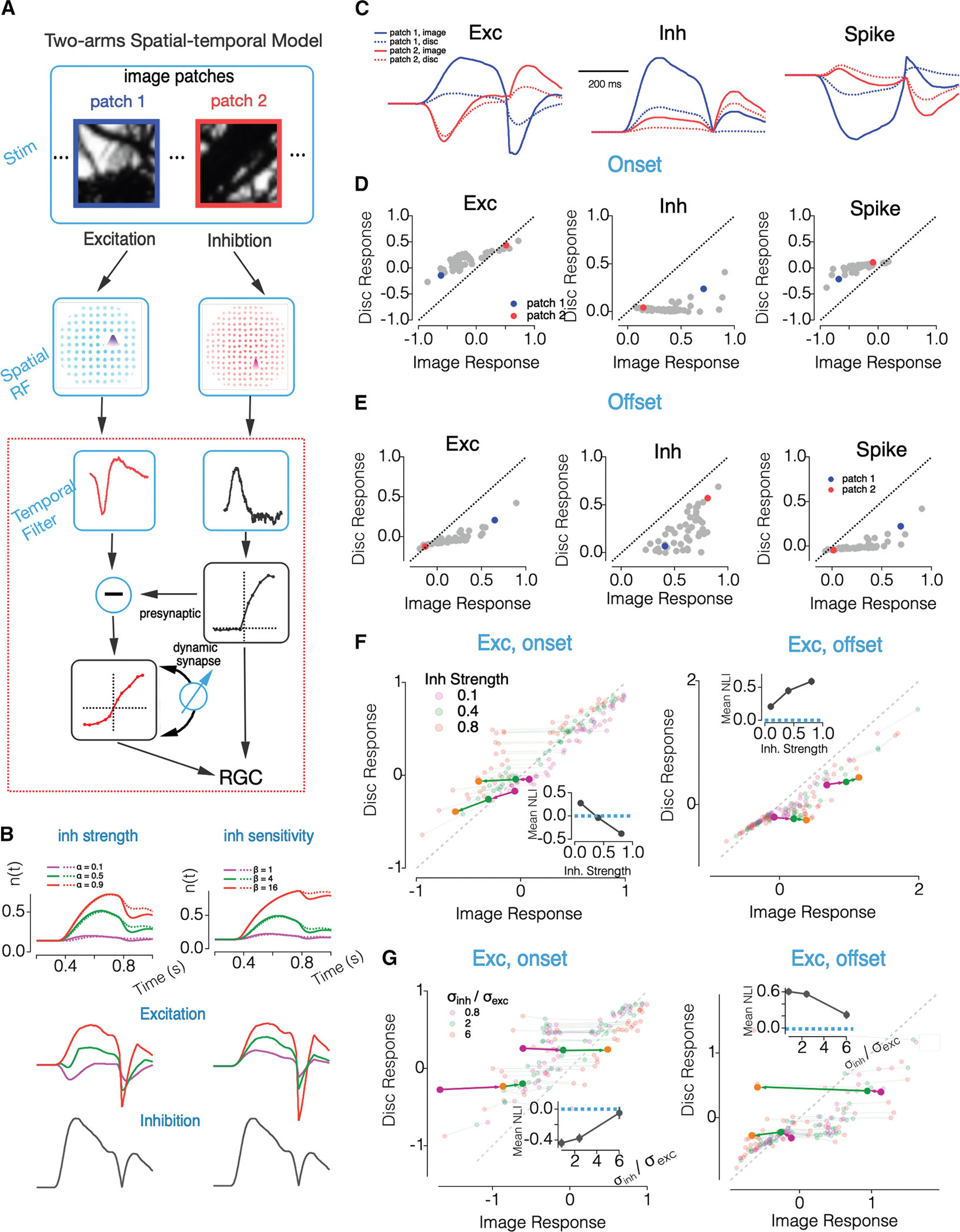
Spatial-temporal dynamics of excitation/inhibition balance mediates homogeneity preference in the retina (A) Schematic of the two-stream spatial-temporal model. Image patches (top) are processed through parallel excitatory and inhibitory pathways with distinct spatial receptive fields (middle row). Both streams then pass through temporal filters. The inhibitory pathway modulates the excitatory pathway through presynaptic inhibition, which operates via a dynamic synapse with depression (blue feedback arrow). The final output integrates these pathways to form the retinal ganglion cell response. (B) Dynamic simulations of the temporal-only model (dashed rectangle in A) showing vesicle occupancy rate and excitatory output under different parameter conditions in response to a flashed grating (as in Figure 3). (Left) Vesicle occupancy dynamics (n(t)) with varying presynaptic inhibition strengths (α = 0.1, 0.4, 0.8). (Right) Vesicle occupancy dynamics with varying inhibition sensitivity values (β = 1, 4, 8). Corresponding excitatory outputs and inhibitory traces are shown below each condition. (C) Example conductance response traces showing excitatory conductance trace (left), inhibitory conductance trace (middle), and spike responses (3Exc-Inh, right) to two natural image patches (solid lines) and their equivalent homogeneous discs (dotted lines). The spatial structure in patch 1 (blue) and patch 2 (red) evokes distinct patterns of excitation and inhibition. (D) Comparison of onset responses to natural image patches versus their equivalent discs for excitation (left), inhibition (middle), and spike output (right). Inhibition strongly prefers structured images (points below unity line), while excitation prefers homogeneous discs (points above unity line). (E) Similar to (C), but showing offset responses after stimulus termination. (F) Simulation results showing how inhibitory strength modulates spatial integration. Scatterplots compare image versus disc responses at stimulus onset (left) and offset (right) with different inhibitory strengths (0.1, 0.4, and 0.8). Responses represent area sums (unitless) normalized to the maximal response during stimulus onset across all conditions. Semi-transparent points represent individual image patches, while thick colored lines show responses for a few example patches. Insets show how the mean nonlinearity index (NLI) changes with inhibitory strength. (G) Same as (F), but for inhibitory/excitatory subunit size ratio (0.8, 2, and 4). Insets show how the mean NLI changes with the subunit size ratio.

Our analyses identified two key mechanisms underlying homogeneity preference in OffT αRGCs: (1) integration of narrowly tuned inhibitory inputs and broadly integrating excitatory inputs and (2) presynaptic inhibition at bipolar terminals that enables recovery from chronic synaptic depression and a corresponding large increase in excitatory input at the offset of spatially structured images. We next sought to develop a unified model that integrates these complementary mechanisms (Figure 8).

Our model builds on a hybrid linear-nonlinear (LN) framework (Figure 8A): the excitatory pathway is a dynamic LN model, in which the slope of the output nonlinearity—reflecting synaptic gain—is multiplicatively scaled by the vesicle occupancy (n), which evolves according to a depletion-and-recovery process. This allows presynaptic inhibition to directly modulate gain by reducing vesicle release rates (see STAR Methods). The inhibitory pathway is implemented as a conventional static LN model, without vesicle dynamics. This mechanistic model is not unique, but it allowed us to explore the interaction between short-term synaptic plasticity and presynaptic inhibition.

We started with a reduced temporal-only implementation (Figure 8B; Figure S5; also see STAR Methods) that focuses on core temporal dynamics. This simplified temporal model tracks excitatory and inhibitory responses across two visual hemifields receiving either matching or opposing contrast inputs, allowing us to isolate temporal mechanisms in response to a flashed grating (as in Figure 3) in a more constrained parameter space before extending to the full spatial-temporal domain. The two-hemifield model successfully replicates two characteristic features of OffT αRGC responses to a flashed grating: suppression of excitatory conductance during stimulus onset followed by rebound excitation at offset (Figure 8B). These effects emerge directly from the presynaptic suppression of vesicle release during stimulation, followed by enhanced release once inhibition is relieved at grating offset. Both the suppression and rebound scale predictably with the strength of presynaptic inhibition strength (*α*) and its sensitivity parameter (*β*). A higher *β* increases the coupling between inhibition and vesicle dynamics, making synaptic output more sensitive to small changes in inhibitory input (Figure 8B; see STAR Methods). Our model also captures other temporal signatures of OffT αRGC responses to additional stimuli, including paired-pulse facilitation and phase-shifted responses to contrast-reversing gratings (see Figure S5).

The full spatial-temporal model (Figure 8A) adds the distinct spatial properties of excitatory and inhibitory subunits observed experimentally (Figure 4F; Table S1). Excitatory and inhibitory subunit radii were set directly from the half-maximal F2 bar-width estimates in Figure 4F. The outputs of these subunits were then pooled by a Gaussian weighting function whose spatial scale (σ) was defined by the RF center size obtained from difference-of-Gaussians fits to expanding-spot responses (Figure S3; Table S1). This model successfully reproduced both suppression of onset responses and enhancement of offset responses to structured natural images (Figures 8C–8E). These effects emerged from the spatially restricted presynaptic inhibition, which suppresses excitatory drive to structured stimuli and by doing so, permits recovery of excitatory synaptic gain. Consistent with our pharmacological experiments (Figure 5), reducing presynaptic inhibition attenuated the predicted homogeneity preference (Figure 8F). Increasing inhibitory subunit size similarly reduced the model’s preference for homogeneous stimuli (Figure 8G). Replicating the observed homogeneity preference also depended on nonlinear properties of both excitatory and inhibitory inputs (Figure S6).

This spatial-temporal framework captures the core computations shaping OffT αRGC responses: structured stimuli activate inhibitory subunits that suppress excitation and permit recovery of synaptic gain, while transitions to homogeneity reveal enhanced excitation through disinhibition and gain facilitation. This model provides a mechanistic basis for understanding how nonlinear spatial integration and history-dependent gain interact to shape retinal output under naturalistic conditions.

## DISCUSSION

OffT αRGCs exhibit unexpected responses to spatial stimuli—responding more strongly to homogeneous stimuli than to those with spatial structure (Münch et al.^19^; see also Bölinger and Gollisch^20^). This is unlike the responses of most RGCs, which are enhanced by spatial structure due to nonlinear RF subunits. The unusual spatial selectivity of the OffT αRGCs suggests that mechanisms not captured by the canonical view of retinal processing shape their responses. Here, we identified two circuit mechanisms that together can account for OffT spatial selectivity. First, synaptic inhibition was tuned to finer spatial scales than excitation, causing a preferential cancellation of responses to fine spatial structure. Second, the excitatory synapses made onto OffT αRGCs exhibited strong short-term synaptic plasticity and the resulting modulation of synaptic gain by presynaptic inhibition amplified responses at the offset of spatial structured stimuli.

### Non-canonical roles of inhibition in spatial integration

The stimulus selectivity of many sensory neurons is shaped by interactions between excitation and inhibition. A canonical form of such interactions is lateral inhibition—in which inhibition is tuned to a broader set of stimuli than excitation and hence can sharpen tuning by suppressing responses to non-optimal stimuli.^21,22,41^ Another canonical form is feedforward inhibition, in which an extra synapse in the inhibitory pathway causes a short temporal delay between excitation and inhibition and a corresponding brief time window in which excitation can dominate the response.^3,42–44^ Both of these forms of inhibition are prominent in retinal circuits.

Crossover inhibition from the On pathway onto Off αRGCs—mediated primarily by A2 amacrine cells—has also been well established.^19,35,45^ Previous studies showed that this inhibition can suppress responses of transient Off αRGCs to spatially structured stimuli and that blocking it can unmask excitation.^19,20^ These findings establish the existence and functional relevance of A2-mediated inhibition, but they do not determine how this inhibition is organized spatially, how it interacts with excitatory synapses, or how it contributes to nonlinear spatial computations that depend on stimulus history.

Our results show that inhibition contributes to spatial computation in a non-canonical manner. Specifically, we found that inhibitory input to OffT RGCs was tuned to finer spatial scales than excitatory input. This difference broadens spatial tuning by allowing inhibition to suppress responses to fine but not coarse spatial structure. In addition, inhibition interacts with short-term plasticity at excitatory synapses, dynamically modulating synaptic gain and producing homogeneity preference at both stimulus onset and offset. These two mechanisms acting in concert can account for the selective responses of OffT cells to spatially homogeneous inputs.

Excitation and inhibition typically share many circuit elements, and hence the stimulus selectivity of inhibition inherits many features from excitation.^5,6,46–48^ The more narrowly tuned inhibition that we observe here requires instead that inhibition originates from circuitry that is largely distinct from excitation, and indeed the preference of OffT RGCs for homogeneous inputs was strongest when excitation and inhibition originated through parallel and distinct retinal circuits—excitation through cone bipolar circuits and inhibition through rod bipolar circuits. This parallel processing allows for differential tuning of excitation and inhibition.

### Synaptic depression as a mechanism for context-dependent processing

History-dependent changes in synaptic strength shape responses in many circuits. For example, depression at excitatory synapses often serves as a gain control mechanism,^25,49^ whereas depression at inhibitory synapses, and the resulting disinhibition, can produce facilitation.^16,50^ These effects occur through a variety of synaptic mechanisms and correspondingly operate on a variety of timescales. Retinal signals are strongly shaped by short-term plasticity due to vesicle depletion and the resulting modulation of synaptic gain.^51–54^

Our findings expand upon this framework, illustrating how synaptic depression at bipolar terminals, when combined with spatially precise glycinergic inhibition, generates a novel form of spatial selectivity. In the absence of a stimulus, we find that the excitatory synapses onto OffT RGCs are tonically active, and correspondingly operate at low gain. Suppression of this tonic activity allows recovery of synaptic gain and large responses to subsequent stimuli. Presynaptic inhibition is a key source of such suppression, and this contributes to the homogeneity preference of these cells. Specifically, the onset of spatially structured stimuli elicits strong presynaptic inhibition, which suppresses vesicle release. This allows vesicle replenishment and enhances the excitatory responses generated at a transition from spatially structured to homogeneous stimuli.

Interestingly, excitatory inputs to OffS cells exhibit a similar level of tonic activity but do not show a recovery of gain following suppression of this tonic activity. Thus, different properties of different bipolar synapses shape responses and contribute to diversification of RGC responses. A similar difference in short-term plasticity at different bipolar cell synapses has been described for On-transient versus On-sustained RGCs.^55^ On-transient cells exhibit more pronounced synaptic depression—consistent with smaller or more rapidly depleting vesicle pools at presynaptic bipolar terminals—whereas On-sustained cells maintain higher baseline release with less depletion during prolonged stimuli.

### Limitations

A limitation of the present study concerns the relationship between functional measurements of presynaptic inhibition and existing connectomic data describing A2 amacrine cell output onto Off bipolar cell types. Functionally, we find a strong asymmetry between OffT and OffS αRGC pathways: glycinergic inhibition from A2 amacrine cells suppresses excitatory drive and produces pronounced rebound excitation at OffT excitatory synapses, whereas similar effects are not observed in OffS cells. This functional asymmetry appears paradoxical in light of connectomic data showing that the majority of A2 glycinergic output synapses in the Off lamina are made onto type 2 bipolar cells, which are the principal excitatory inputs to OffS αRGCs. OffT cells receive input predominantly from type 3a, 3b and 4 bipolar cells, which receive fewer A2 synapses.^56,57^ Several issues may help explain this apparent paradox. Bipolar synaptic release reflects a combination of excitatory input to the bipolar dendrites and inhibitory input to the synaptic terminal; type 2 bipolar cells may indeed receive stronger inhibitory input from A2 amacrine cells that is offset by stronger excitatory dendritic input. Further, anatomical connectivity may not predict functional influence: A2 amacrine cells contact all of these Off-bipolar subtypes in the inner plexiform layer,^58,59^ but the magnitude and dynamics of presynaptic inhibition may not be well constrained by connectomic reconstructions alone. Further studies of the intrinsic properties of Off-cone bipolar subtypes and their synaptic interactions with A2 amacrine cells are needed to elucidate the root cause of this asymmetry.

A second limitation is that we lack a complete picture of the functional role of OffT αRGCs, particularly across light levels. At photopic light levels, some of the same mechanisms we characterize here provide sensitivity to looming stimuli,^17,19^ and OffT αRGCs are necessary for looming-evoked defensive behaviors.^60^ At these light levels, OffT αRGC responses, like those of many other RGC types, are enhanced by spatial structure. As light levels decrease, however, the spatial sensitivity of these cells shifts to a preference for spatially homogeneous rather than spatially structured stimuli. Future studies will be needed to directly link light-level-dependent changes in OffT αRGC output to downstream decoding and behavior under controlled manipulations of illumination and visual context.

## RESOURCE AVAILABILITY

### Lead contact

Further information and requests for resources should be directed to the lead contact, Fred Rieke (rieke@uw.edu).

### Materials availability

This study did not generate any new materials.

### Data and code availability

- Data from this study are available from the lead contact upon reasonable request.
- Analysis codes used in the manuscript are available from GitHub (https://github.com/chrischen2/spatialIntegration), with a DOI version record through Zenodo at https://zenodo.org/records/18491916. All analysis code was written and tested in MATLAB R2024b. All visual stimulus code (Stage-VSS, Symphony-DAS) was written in MATLAB R2016b.
- Any additional information required to reanalyze the data reported in this paper is available from the lead contact upon request.

## STAR★METHODS

### EXPERIMENTAL MODEL AND STUDY PARTICIPANT DETAILS

Experiments were conducted on whole-mount retinas from dark-adapted (overnight) wild-type (C57/BL6 or sv-129, either sex, 1–12 months)) mice using procedures in compliance with the University of Washington’s Institutional Animal Care and Use Committee.

### METHOD DETAILS

#### Electrophysiology

The retina was isolated with Retinal Pigment Epithelium (RPE) and sclera attached and stored in oxygenated (95% O2/5% CO2) Ames medium (Sigma) at 32°C–34°C. For C57/BL6 animals, dorsal-ventral orientation was determined using the natural landmarking on the posterior eyecup. In sv-129 animals, which lack a reliable posterior scleral landmark, dorsal-ventral orientation was instead labeled prior to enucleation by marking the cornea with a surgical skin marker (or permanent marker) after briefly drying the corneal surface; this mark was then used to maintain orientation during dissection.Isolated retinas were flattened onto poly-L-lysine slides before being transferred under the microscope for recording and were continuously perfused with Ames medium at a flow rate of approximately 8 mL/min. For cell-attached and whole-cell recordings, retinal neurons were visualized and targeted using infrared light (>950 nm).

Voltage-clamp recordings were obtained using pipettes (for RGCs, 2–4 MΩ) filled with an intracellular solution containing (in mM) the following: 105 Cs methanesulfonate, 10 TEA-Cl, 20 HEPES, 10 EGTA, 2 QX-314, 5 Mg-ATP, 0.5 Tris-GTP, and 0.1 Alexa 750 hydrazide (~280 mOsm; pH ~7.3 with CsOH). Current clamp recordings for A2 amacrine cells were conducted with pipettes ~8 MΩ using an intracellular solution containing (in mM) the following: 123 K-aspartate, 10 KCl, 10 HEPES, 1 MgCl2, 1 CaCl2, 2 EGTA, 4 Mg-ATP, 0.5 Tris-GTP, and 0.1 Alexa 750 hydrazide (~280 mOsm; pH ~7.2 with KOH). NBQX (10 μm; Tocris) or TTX (0.5 μm; Alamone) was added to the perfusion solution. For whole-cell voltage-clamp recordings, series resistance was monitored continuously throughout each experiment. Only recordings with an initial series resistance <14 MΩ were included for analysis. Across the included dataset, initial series resistance ranged from 3.7 to 14 MΩ (mean ± SEM: 7.66 ± 0.43 MΩ). Series resistance remained stable over the course of recordings, with final values of 8.9 ± 0.55 MΩ (range: 3.7–17.6 MΩ). Series resistance was compensated online by 50–75% for all voltage-clamp recordings.

For Off αRGC voltage-clamp recordings, holding potentials used to isolate inhibitory and excitatory synaptic currents were determined empirically for each cell using large contrast steps from a defined mean background, as in prior work validating effective separation of synaptic conductances in αRGCs.^3^ To set the holding potential for isolating inhibitory currents, we delivered a −90% contrast step and adjusted the holding potential until the inward current at flash onset was minimized or eliminated. Conversely, to set the holding potential for isolating excitatory currents, we delivered a +90% contrast step and adjusted the holding potential until the inward current attributable to residual inhibitory conductance (“leak” from the inhibitory channel) was minimized or eliminated. Reversal potentials were checked periodically during recordings using these contrast steps to verify stability of synaptic isolation over time.

Absolute voltage values were not corrected for liquid junction potentials (*K*^+^ based = −10.8 mV; *Cs*^+^ based = −8.5 mV).

#### Visual stimuli

Stimuli were generated, and visual response data was acquired using custom-written stimulation and acquisition software packages Stage (http://stage-vss.github.io) and Symphony (http://symphony-das.github.io), respectively. These stimuli were displayed at a frame rate of 60 Hz on an OLED microdisplay monitor (800 × 600 pixels, eMagin), with a focus through a 10× objective, directly onto the photoreceptors. The configuration resulted in a resolution of either 1.8 μm/pixel or 1 μm/pixel at the retina, respectively, for two different recording rigs used. Stimuli were calibrated using the measured monitor optical power output, the spectral content of the monitor, mouse photoreceptor spectral sensitivity, and a collecting area of 0.5 *μm*^2^ for mouse rods. Unless otherwise specified, mean light levels produced ~100 isomerizations per rod per second (R*/rod/s).

At the beginning of each recording, the cell’s receptive field center was identified using a split-field contrast reversing grating stimulus at 2Hz and 90% spatial contrast. The stimuli were adjusted until the F2 (double frequency) response cycles were equalized, and conducted along both horizontal and vertical axes. This centering process was repeated for each type of response measured from a given cell (i.e., spike output, excitatory synaptic input, inhibitory synaptic input). All subsequent stimuli were aligned with the receptive field center. To determine the size of the receptive field center or surround of the RGC, a classical difference of the Gaussian model was applied to the area summation curve in response to uniform spots of varying diameters. The model is described as follows:

$$R=K_{c}\left(1-exp\left(-\frac{r^{2}}{2\sigma_{c}}\right)\right)-K_{s}\left(1-exp\left(-\frac{r^{2}}{2\sigma_{s}}\right)\right)+R_{0}$$

Where as R is the response of the cell, $R_{0}$ is the spontaneous activities, r is the radius of the expanding spot, $\sigma_{C}$ and $\sigma_{S}$ are the radius of the center, and surround, respectively, and $K_{C}$ and $K_{S}$ describe the scaling strength of the center and surround components respectively.

Naturalistic stimuli were generated using images from the van Hateren natural image database.^61^ For each source image, we applied a single global scaling such that the brightest point in the entire image (from which the image patches were derived) was set as the highest monitor intensity. Natural image patches were generated from random samples of a given image. Importantly, this scaling was applied at the level of the full image and did not normalize the mean luminance (or contrast) of individual patches. For each image, we randomly sampled 10,000 candidate patches. The background intensity when presenting natural image patches to cells was similarly defined as the mean intensity across the full source image from which the patches were drawn. Subsets of natural image patches were selected as stimuli, as described before 1.^18,30^ In short, we incorporated the nonlinear subunit model previously developed for classifying image patches by their response nonlinearity. This involved comparing the difference in responses between spatially linear versus nonlinear RF models. We then selectively sampled across the full spectrum of image patches to present a balanced range, from highly structured patches with strong spatial contrast to relatively homogeneous patches with minimal spatial contrast. The disc diameter (and the matched image-patch aperture) was set for each cell based on the RF-center size, defined as the 2σ width of the RF-center Gaussian obtained from difference-of-Gaussians fits to expanding-spot responses. This approach enabled us to efficiently investigate the entire spectrum of spatial contrasts found in natural images in a single experiment. Patch-level luminance and spatial statistics are now quantified and summarized in Figure S7.

To construct the linear equivalent disc stimulus, we computed the weighted average pixel intensity of each image patch with a circular Gaussian function; Gaussian weights were determined for each recorded cell from responses to spots of a number of sizes. The weights for the Gaussian function were derived from each recorded cell’s response to differently sized spots. The Gaussian function’s width, defined by its two-standard deviation span, corresponded to the diameter of the aperture overlaying the image, which matched the measured size of the RF center. These natural image patches were then displayed at a retinal scale of 6.6 μm/pixel.

The linear equivalent disc approach assumes radially-symmetric receptive fields, but real RGCs often deviate from this assumption. Several observations indicate that this discrepancy does not confound our results. First, primate On parasol RGCs respond similarly to natural image patches and linear equivalent discs, while Off parasol RGCs respond quite differently.^18^ Both cell types have similarly shaped RFs.^62^ Second, OffT αRGC spatial integration changes from a preference for homogeneity to a preference for spatial structure with increasing light level (Figure 6), which is difficult to explain as a systematic bias in estimating RF properties.

One concern about interpretation of the contrast-reversing grating experiments is that spatially offset excitatory and inhibitory receptive fields can be converted into apparent timing offsets, particularly for moving stimuli 6.^63,64^ However, in our experiments the gratings were stationary (either periodically contrast-reversed or flashed), and stimulus alignment was controlled independently for each spike, excitatory-current, and inhibitory-current recording by centering a split-field contrast-reversing grating on the receptive-field (RF) center. The resulting offsets between recording sessions were small (mean ~6 μm, rarely exceeding ~10 μm; Figure S8), and thus were minor relative to the RF center size of OffT αRGCs. Moreover, the inhibitory-leading–excitatory phase relationship was observed consistently across a broad range of bar widths (~5–160 μm, Figure S9), arguing against an explanation based on a particular spatial scale or on the precise positioning of grating edges within the RF. Grating stimuli were presented within a circular aperture (~320 μm diameter), further limiting sensitivity to large-scale spatial misalignment.

#### Cell identification and selection criteria

We identified alpha RGCs based on their characteristic large soma size and distinctive spike responses to light steps.^65^ RGCs recordings were performed with retinal pigment epithelium (RPE) attached to preserve natural retinal function. To ensure consistent preparation quality and light sensitivity across experiments, we screened each retinal preparation prior to OffT αRGC recordings by measuring the response of an On-sustained αRGC to a step from complete darkness to ~0.5 R*; only preparations exhibiting clear, robust responses were used for subsequent recordings.

#### Analysis

##### Center-surround subunit model

The center-surround subunit model was implemented in MATLAB (adapted from^30^), to simulate the responses of retinal ganglion cells (RGCs) to grating stimuli of varying widths (Figure 4). The receptive field (RF) of each subunit was modeled as a difference of Gaussians (DoG) with a variable inhibitory subunit center size $\sigma_{\mathrm{inh}}$ and surround ${\sigma_{\mathrm{inh}}}^{\mathrm{surround}}$ and the strength of the subunit surround (δ) was varied relative to the center (0.2 for weak surround, 0.5 for moderate surround, and 0.9 for strong surround, Figure 4). The equation for each subunit’s RF is:

$$RF(x)=exp\left(-\frac{x^{2}}{2\sigma_{inh}^{2}}\right)-\delta \cdot exp\left(-\frac{x^{2}}{2{\sigma_{inh}^{\mathrm{surrount}}}^{2}}\right)$$

where x represents spatial position. Subunit responses were weighted by their position within the overall RF center (σ=50μm). Bar stimuli of varying widths were generated and circularly shifted across trials to simulate variability. This setup allowed us to examine how varying the subunit surround strength and size influenced spatial integration in the RGCs.

##### Dynamic synapse model

We implemented a hybrid linear–nonlinear (LN) model that captures the core circuit dynamics we observed experimentally. The model incorporates two key elements: (1) an excitatory pathway governed by short-term synaptic depression, reflecting the bipolar cell terminals that undergo activity-dependent modulation, and (2) a static inhibitory pathway representing the A2 amacrine cell inputs. In this formulation, the excitatory output is regulated by a vesicle occupancy variable (representing available neurotransmitter vesicles at bipolar cell terminals) that changes according to synaptic depression dynamics.

The model accommodates both spatiotemporal (Figure 8A) and hemifield-based stimuli (Figure 8B). In the spatiotemporal configuration, each excitatory subunit integrates visual input across space and time, while receiving pooled inhibition from neighboring inhibitory subunits. For hemifield stimuli (Figure 8B),—spatial structure is collapsed, and each hemifield contains one excitatory and one inhibitory subunit. In this case, each excitatory subunit is modulated by the combined inhibition from both inhibitory subunits. (Model code is available at: https://github.com/chrischen2/spatialIntegration.git).

##### Inhibitory subunit: Static LN model

Each inhibitory subunit j located at position $y_{j}$ processes stimulus input (x,t) through a spatial-temporal linear kernel $k_{\mathrm{inh}}(x,\tau )$, followed by a rectifying nonlinearity:

$$I_{i}(t)={\left[\int_{0}^{T}\;\int_{-\infty }^{\infty }\;k_{inh}\left(x-y_{j,\tau }\right)S(x,t-\tau )dxd\tau \right]}_{+}$$

In the spatial-temporal model, the spatial receptive field of inhibitory subunits ${RF}_{\mathrm{inh}}(x)$ is defined by the center-surround structure (with strong and local surround) characterized in Figure 4.


$$k_{inh}(x,\tau )=R_{inh}(x)\cdot T_{inh}(\tau )$$

Where $T_{inh}(\tau )$ is the temporal filter of inhibitory subunits, which is extracted from experimental data. ${\left[\cdot \right]}_{+}$: half-wave rectification (threshold-linear nonlinearity). $R_{inh}(x)$ is the spatial receptive field of inhibitory subunits, modeled as difference of Gaussian (as Figure 4) and defined as equation below:

$$R_{inh}(x)=exp\left(-\frac{x^{2}}{2\sigma_{inh}^{2}}\right)-\delta \cdot exp\left(-\frac{x^{2}}{2\sigma_{inh,\mathrm{surround}\;}^{2}}\right)$$

where $\sigma_{\mathrm{inh}}$ and $\sigma_{\mathrm{inh},\;surround}$ denote the center and surround spatial scales of inhibitory subunit, respectively, and δ is the relative surround strength (dimensionless, 0<δ≤1). These parameters were chosen to match the spatial inhibitory receptive fields observed experimentally and illustrated in Figure 4.

In the hemifield-based temporal model (used in Figure 8B), spatial integration is omitted and each hemifield is represented by a single inhibitory subunit. In that case:

$$I_{i}(t)={\left[\int_{0}^{T}\;k_{inh}(\tau )\cdot S_{i}(t-\tau )d\tau \right]}_{+}$$

Here, $l_{j}(t)$ is a static inhibitory signal and does not undergo dynamic synaptic changes. $S_{j}(t)$ is the stimulus presented to hemifield j, where j∈{left,right} indicates the hemifield.

##### Excitatory subunit: Dynamic LN model with presynaptic inhibition

Excitatory drive for each excitatory subunit i, located at $x_{i}$, receives linearly filtered input via an excitatory kernel $k_{\mathrm{exc}}(x,\tau )$, followed by presynaptic inhibition with strength factor α :

$$E_{i}^{raw}(t)=\int_{0}^{T}\int_{-\infty }^{\infty }k_{exc}\left(x-x_{i,\tau }\right)S(x,t-\tau )dxd\tau$$

This signal is modulated by presynaptic inhibition, pooled from nearby inhibitory subunits using a Gaussian weighting function:

$$I_{i}^{\mathrm{eff}\;}(t)=\alpha \cdot \sum_{j}w\left(X_{i},y_{j}\right)\cdot I_{i}(t)$$

Where $w\left(x_{i},y_{j}\right)$ defines the spatial influence of inhibitory subunit j on excitatory subunit i, and α is a global inhibition strength factor.


$$w\left(x_{i},y_{j}\right)=exp\left(\;-\;\frac{{\left(x_{i}-y_{j}\right)}^{2}}{2\sigma_{inh}^{2}}\right)$$

The presynaptically modulated excitatory input is passed through a static piecewise linear nonlinearity Φ(⋅) defined as:

$$E_{i}(t)=\Phi \left(E_{i}^{\mathrm{raw}}(t)-l_{i}^{\mathrm{eff}}(t)\right)$$

In our model, excitation output nonlinearity Φ(x) is given by:

$$\Phi (x)=\left\{\begin{array}{cc} \gamma \cdot x, & x<0\\ x, & x\geq 0 \end{array}\right.$$

where γ, or the rectification ratio, ∈(0,1) is a constant that scales the slope of the negative portion of the input.

Then, the positive portion of this signal is dynamically scaled by the vesicle occupancy ni(t)∈[0,1], which captures short-term synaptic depression. The final output of subunit i is:

$${\tilde{E}}_{i}(t)=n_{i}(t)\cdot max\left(0,E_{i}(t)\right)+R_{0}$$

This formulation scales output by the availability of synaptic vesicles. The dynamics of vesicle occupancy are given by:

$$\frac{dn_{i}}{dt}=\left(1-n_{i}\right)\cdot K_{rec}-b\cdot K_{rel}-n_{i}\cdot {\tilde{E}}_{i}(t)$$

where $K_{\mathrm{rec}}$ and $K_{\mathrm{rel}}$ are vesicle recovery and release rates, and b is a gain constant.

In the temporal (hemifield) model, each hemifield contains one excitatory and one inhibitory subunit, and inhibitory pooling reduces to:

$$I_{i}^{eff}(t)=\alpha \cdot \left(I_{L}(t)+I_{R}(t)\right)$$

##### Pooling onto the ganglion cell

In the spatiotemporal model, the total excitatory and inhibitory drive to the ganglion cell is obtained by spatially weighted summation across subunits. Let $W_{\mathrm{exc}}\left(x_{i}\right)$ and $W_{\mathrm{inh}}\left(y_{j}\right)$ be Gaussian spatial weights centered on the soma:

$$E_{\mathrm{total}}(t)=\sum_{i}\;W_{\mathrm{exc}}\left(X_{i}\right)\cdot {\tilde{E}}_{i}(t)$$

$$I_{\mathrm{total}}(t)=\sum_{i}\;W_{\mathrm{inh}}\left(y_{i}\right)\cdot I_{j}(t)$$

For hemifield-based stimuli (e.g., step and pair-pulse paradigms), each hemifield consists of one excitatory and one inhibitory subunit. Spatial weighting is omitted, and the total excitatory and inhibitory drives computed as the average of the subunit outputs.

##### Model parameters

For all simulations, constant parameters—such as the vesicle recovery and release rates ($K_{\mathrm{rec}}$ and $K_{\mathrm{rel}}$), release gain (b). The rectification ratio γ, which defines the slope of the subthreshold (negative) region of the excitatory nonlinearity Φ(x), was constrained using experimental data. Specifically, we used values derived from nonlinearities measured in Off-transient alpha RGCs stimulated with temporal Gaussian noise and sinusoidal modulations under mesopic conditions (see Table S1).

To further constrain the model,the initial vesicle occupancy $n_{0}$ was not treated as a free parameter but was inferred directly from experimental measurements of baseline excitation.


$$n_{0}=\frac{1}{1+b\cdot E_{0}\cdot \frac{K_{rel}}{K_{rec}}}$$

This expression arises from setting $\frac{dn}{dt}=0$ in the vesicle dynamic equation under constant baseline drive and we also assume in the initial steady state baseline inhibition is zero. By using this constraint, the model ties the initial condition $n_{0}$ directly to measurable physiological quantities.

The qualitative dynamics we study arise robustly from the model structure rather than specific parameter tuning.

## Supplementary Material

1

SUPPLEMENTAL INFORMATION

Supplemental information can be found online at https://doi.org/10.1016/j.celrep.2026.117306.

## Figures and Tables

**KEY RESOURCES TABLE:** 

REAGENT or RESOURCE	SOURCE	IDENTIFIER

Biological samples		

Mouse retina (wild-type 129S1/SvlmJ)	The Jackson Laboratory	RRID:IMSR_JAX:002448

Chemicals, peptides, and recombinant proteins		

Ames	MilliporeSigma	A1420
Strychnine	Sigma	S8753
GABAzine	Sigma	SR-95531
TPMPA	Tocris	1040
L-AP4 (APB)	abcam	120002
LY341495	Tocris	1209

Deposited data		

Experimental data and code	This study	https://zenodo.org/records/18491916

Software and Algorithms		

MATLAB	Mathworks	https://www.mathworks.com/products/matlab.html
Stage	Stage-VSS	https://stage-vss.github.io
Symphony	Symphony-DAS	http://symphony-das.github.io
Igor Pro	Wavemetrics	N/A

## Highlights

- Off-transient retinal ganglion cells show a preference for spatially homogeneous inputs
- Inhibition cancels excitatory responses to fine spatial structure
- Strong short-term synaptic plasticity depends on the spatial structure of the input
- These two mechanisms work together to create selectivity for homogeneous stimuli
