# Supplementary material for: Spatially local inhibition and synaptic plasticity together enable dynamic, context-dependent integration of parallel sensory pathways
Source: Cell Rep. Author manuscript; Available in PMC 2026 Jul 3. (PMC13330012; doi:10.1016/j.celrep.2026.117306)
Supplement: 1 [file NIHMS2180955-supplement-1.pdf]

Cell Reports, Volume *45*

Supplemental information

Spatially local inhibition and synaptic plasticity together enable dynamic, context-dependent integration of parallel sensory pathways

Qiang Chen and Fred Rieke

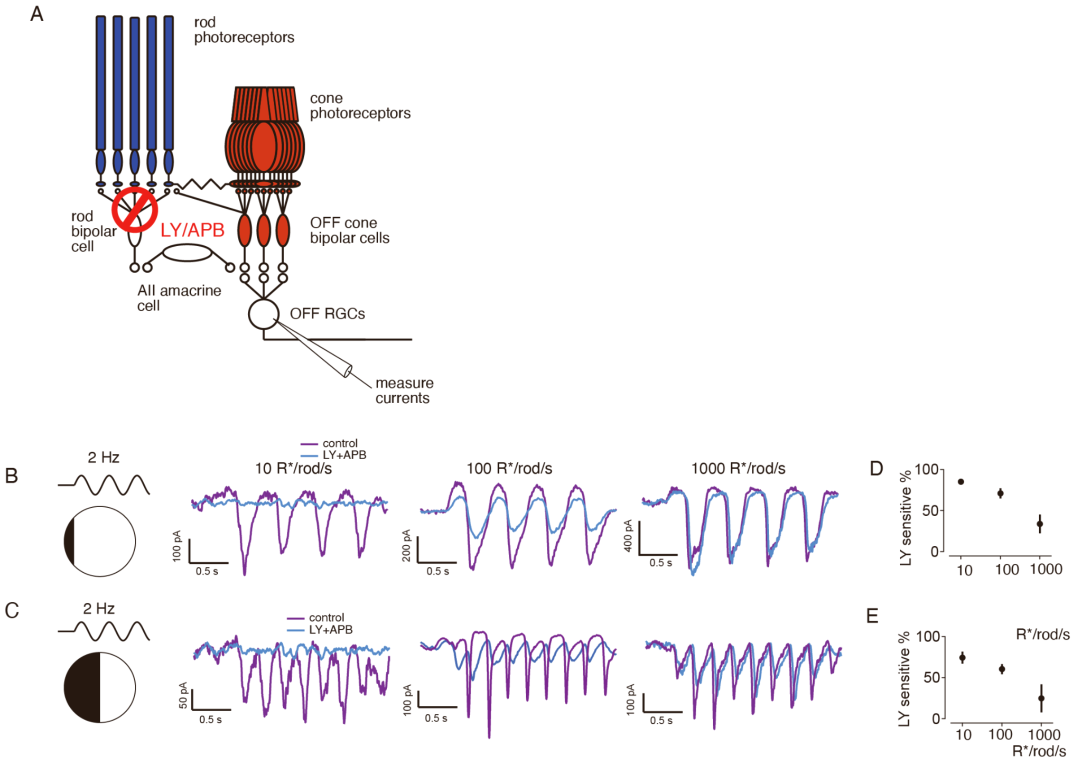

Supplementary Figure 1. Routing of Rod-derived signals under different light levels.

(A) Schematic of the experimental setup showing pharmacological isolation of rod pathways using LY/APB to block rod bipolar cell signaling in the primary rod pathway.

(B) Example current traces (control, purple; LY+APB treatment, blue) from OffT αRGC in response to a 2 Hz full-field modulating spot at 10 R*/rod/s, 100 R*/rod/s, and 1000 R*/rod/s.

(C) Similar current traces showing OffT αRGC responses to a contrast-reversing grating stimulus.

(D-E) Quantification of the LY/APB-sensitive component of the OffT αRGC responses at different light intensities (R*/rod/s). n=9 cells.

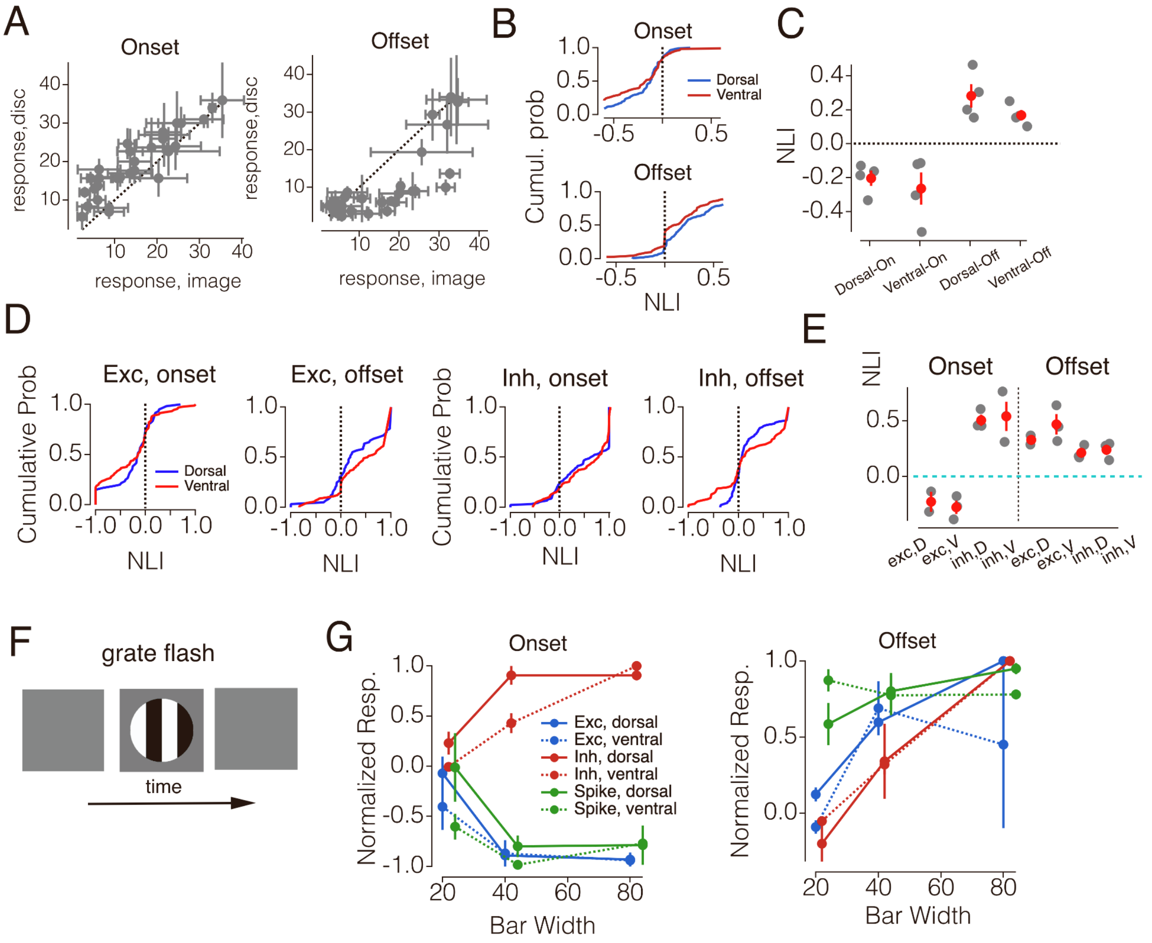

Supplementary Figure 2: Comparison of homogeneity preference in dorsal and ventral OffT cells.

(A) Spatial integration in dorsal and ventral retinal ganglion cells. Example (ventral) scatter plots comparing spike responses to natural image patches versus their linear-equivalent discs for onset (left) and offset (right) responses. Dashed line indicates unity. Error bars represent SEM.

(B) Onset and offset response summary for spike responses. Cumulative distribution of nonlinearity indices (NLI) across all image-patch/disc pairs for dorsal (blue) and ventral (red) cells at onset (top) and offset (bottom).

(C) Summary of mean NLIs for individual cells (gray circles) and population means ± SEM (red circles) for dorsal and ventral regions at onset and offset. (n=4 cells, dorsal; n=4 cells ventral).

(D) Comparison of dorsal vs ventral synaptic inputs. Cumulative distributions of NLI for excitatory (Exc) and inhibitory (Inh) synaptic inputs at onset and offset, comparing dorsal (blue) and ventral (red) populations.

(E) Summary of mean NLIs by cell type and region for onset (left) and offset (right) responses. Individual experiments shown as gray circles; population means ± SEM shown as red circles. Exc, excitatory; Inh, inhibitory; D, dorsal; V, ventral. (n=2 cells Exc, D; n=3 cells, Exc, V; n=3 cells, Inh, D; n=3 cells, Inh, V).

(F) Schematic of the flashed grating stimulus paradigm.

(G) Normalized synaptic and spike responses as a function of bar width for dorsal (solid lines) and ventral (dashed lines) regions at onset (left) and offset (right). Blue: excitatory input; red: inhibitory input; green: spike output. Negative values indicate suppression relative to baseline.

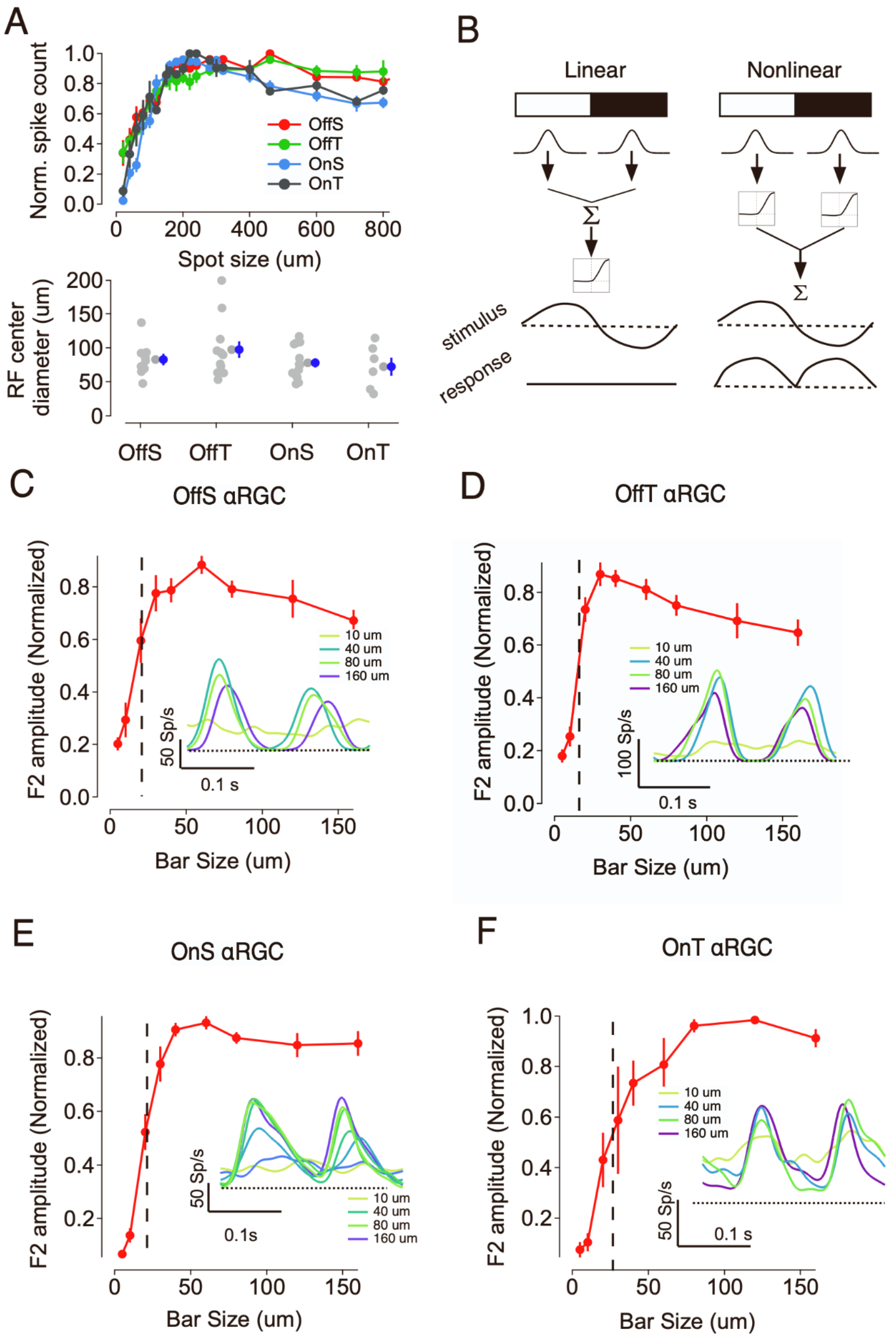

Supplementary Figure 3. Nonlinear subunits in αRGCs receptive field.

(A) Top: Area summation curves for four alpha retinal ganglion cell (αRGC) subtypes: Off-sustained (OffS), Off-transient (OffT), On-sustained (OnS), and On-transient (OnT). The summation curves plot the normalized response amplitude as a function of stimulus diameter

Bottom: Scatter plots show the distribution of RF sizes. Error bars represent mean ± SEM.

(B) Schematic of the contrast-reversing grating used to test for nonlinear subunits in αRGC receptive fields. Nonlinear subunit integration produces frequency-doubled (F2) responses to this stimulus, whereas linear receptive fields do not.

(C-F) Population summary data showing the nonlinear F2 response amplitude as a function of bar width. Inset: cycle average responses in an example αRGC of respective subtype. Vertical dashed lines mark the normalized bar width that elicited the half-maximal F2 response; we define this value as the subunit radius. n=8, 13, 12, 7 cells for OffS, OffT, OnS, and OnT αRGC respectively.

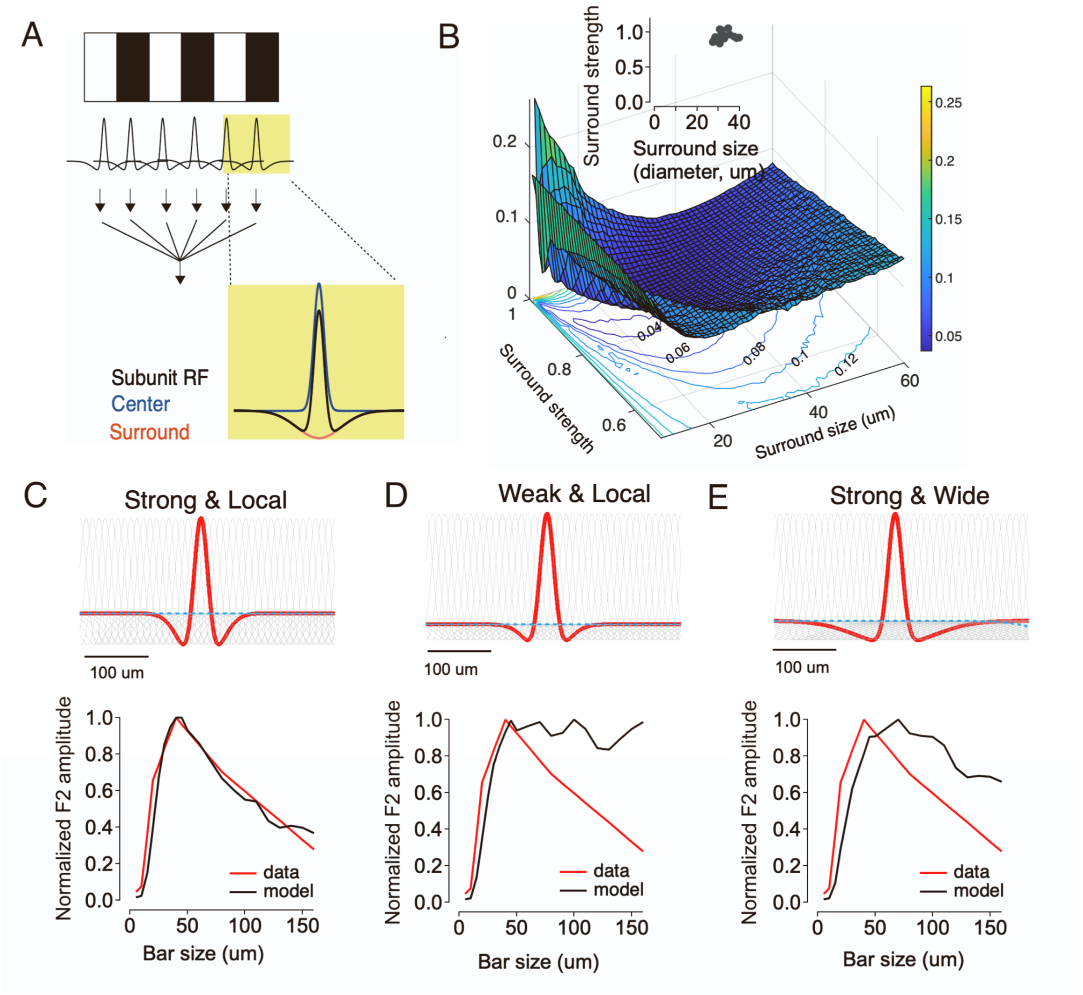

Supplementary Figure 4. Strong and local surround mediates spatial tuning of inhibitory subunits.

(A) Schematic of the center-surround subunit model for inhibitory inputs.

(B) Parameter space showing the effects of surround size and strength on model fit quality.

(C-E) Model fits (black) to experimental data (red) for inhibitory inputs with different surround properties: strong & local (C, surround size 15 μm, surround strength 0.9), weak & local (D surround size 15 μm, surround strength 0.2), and strong & nonlocal (E, surround size 40 μm, surround strength 0.9).

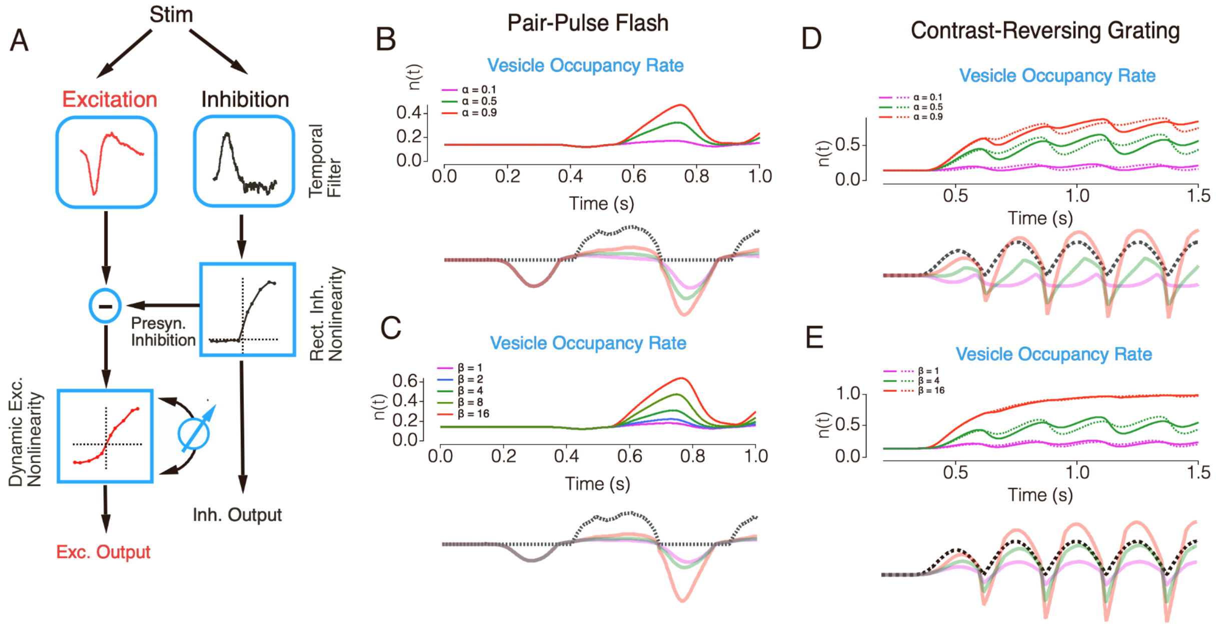

Supplementary Figure 5. Dynamic Synaptic Depression Mediates History-Dependent Excitation/Inhibition Interaction in Retinal Processing

(A) Schematic of the temporal dynamic synapse model showing the excitatory pathway (red) and inhibitory pathway (black). Inhibition modulates excitation through presynaptic inhibition, with inhibitory pathways passing through rectifying nonlinearity while excitatory pathways passing through piecewise nonlinearity. The blue arrow indicates the dynamic regulation of excitatory output through vesicle occupancy.

(B-C) Simulated responses to paired-pulse flash stimuli. Top panels show vesicle occupancy rate (n(t)) and bottom panels show excitatory conductance output (colored lines) along with inhibitory current (dashed black line). (B) demonstrates the effect of varying presynaptic inhibition strength (α) while (C) shows responses with different inhibition sensitivity values (β).

(D-E) Model responses to contrast-reversing grating stimuli.

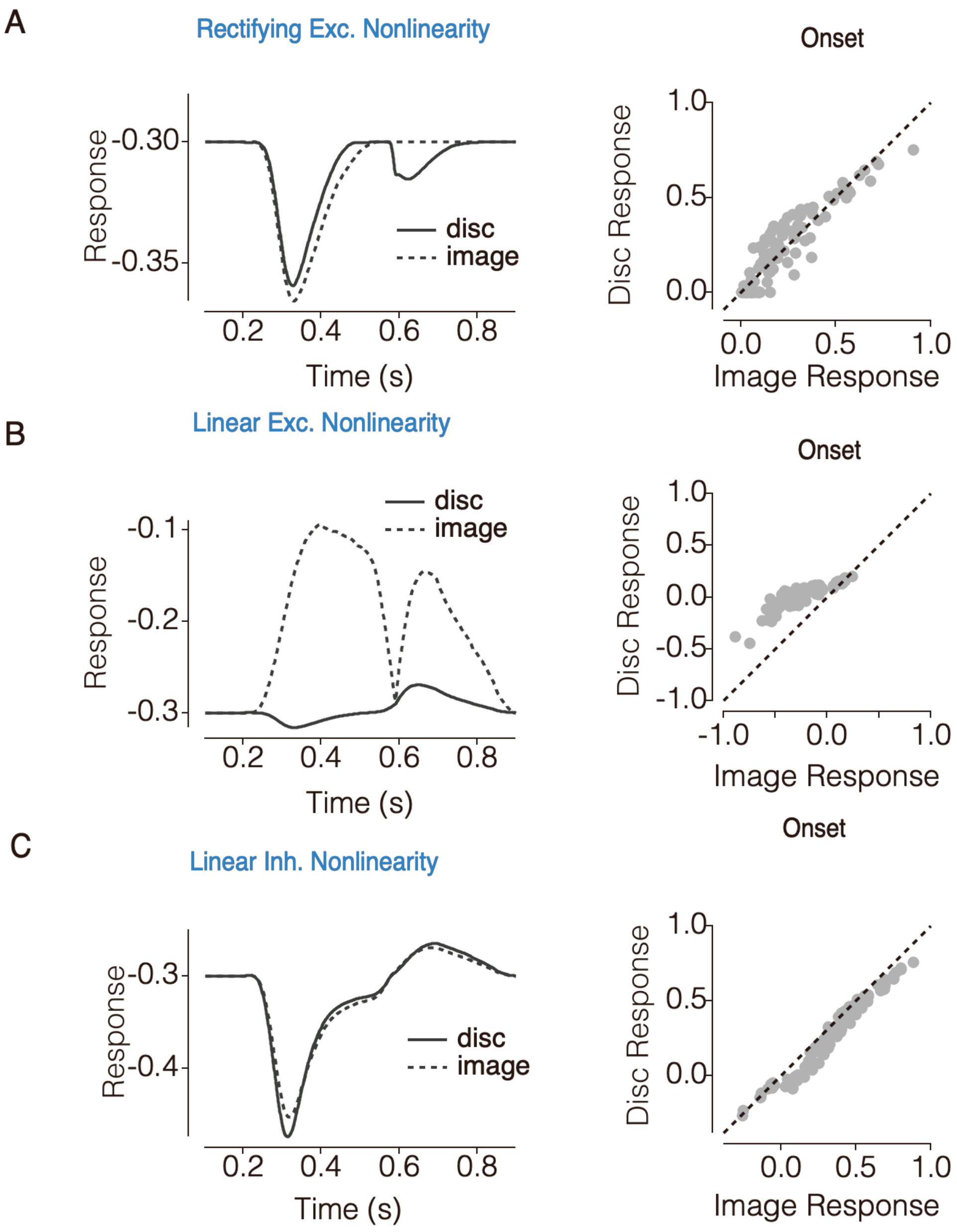

Supplementary Figure 6: Effects of excitatory and inhibitory nonlinearities on spatial integration in the retina

(A) Example response of the model with rectifying excitatory nonlinearity to natural image patches (dashed line) and their corresponding linear equivalent discs (solid line). The left panel shows example response traces over time. The right panel shows the scatter plot of image responses versus disc responses at stimulus onset, with most points clustered along the unity line (dashed), indicating similar responses to both stimulus types.

(B) Response of the model with linear excitatory nonlinearity to the same stimuli. The left panel shows example traces demonstrating suppression of responses to natural images (dashed line) compared to disc stimuli (solid line). The right panel shows the scatter plot of responses at stimulus onset.

(C) Response of the model with linear inhibitory nonlinearity. Left panel shows example traces with similar responses to both natural images (dashed line) and linear equivalent discs (solid line). Right panel shows the scatter plot of responses at stimulus onset, with points clustered along the unity line, indicating that linear inhibition does not generate differential responses to spatial structure.

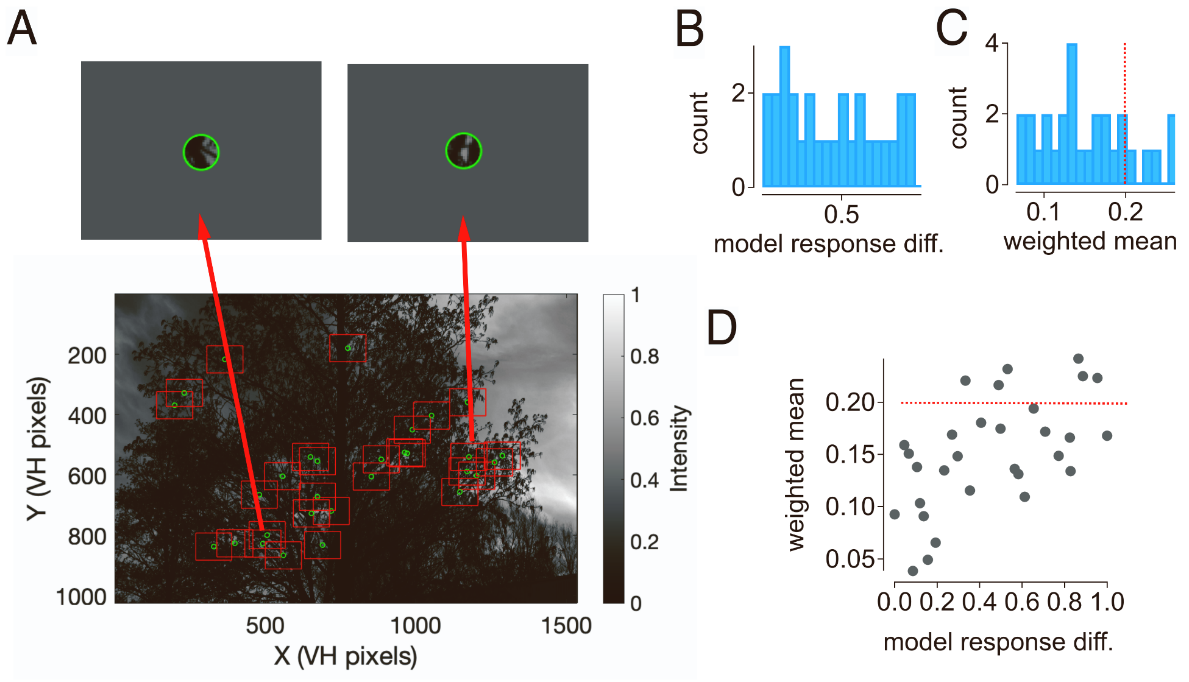

Supplementary Figure 7. Natural image patch sampling and patch statistics.

(A) Example of 30 image patches sampled from a single image in the van Hateren natural image database. Red rectangles indicate patch locations within the source image; coordinates are in van Hateren (VH) pixels. VH pixels were mapped onto the retina at 6.6 µm/pixel. Top panels show enlarged views of two representative patches, displayed through a circular aperture matched to the recorded cell's RF-center size (green circles; ~100 µm diameter in this example). A Gaussian-weighted average within this aperture determined the equivalent intensity for each patch.

(B) Distribution of model response differences (normalized to range 0 to 1) across all sampled patches. The normalized difference between a linear-nonlinear (LN) model and a spatial subunit mode, provides a measure of local spatial structure within each patch.

(C) Distribution of Gaussian-weighted mean intensities across patches. Red dashed line indicates the background intensity (0.199), computed as the mean of the entire source image shown in A, after pixel values were normalized such that the brightest pixel in the source image was set to 1.

(D) Relationship between model response difference and weighted mean intensity for each patch. Red dashed line indicates the background intensity.

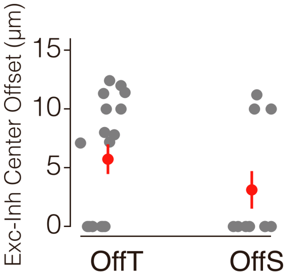

Supplementary Figure 8:Spatial offset between excitatory and inhibitory receptive field centers in Off-type αRGCs.

Quantification of the spatial offset between excitatory and inhibitory receptive field centers for Off-transient (OffT) and Off-sustained (OffS) αRGCs. Individual cells shown as gray circles; population means ± SEM shown as red circles.

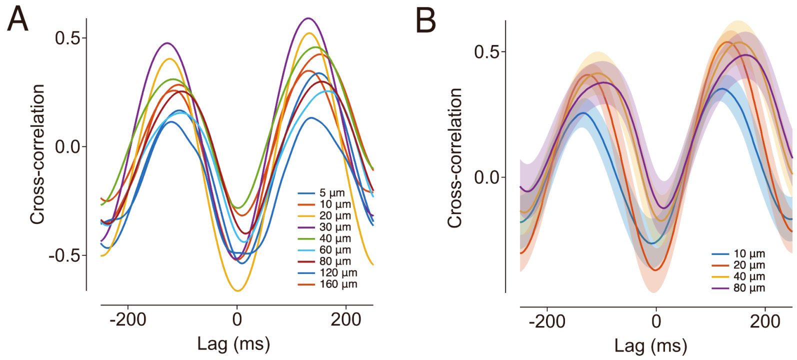

Supplementary Figure 9. Cross-correlation between excitatory and inhibitory synaptic inputs is independent of bar width in OffT αRGCs.

(A) Cross-correlation functions between excitatory and inhibitory currents recorded from a single OffT αRGC, overlaid across different bar widths (5–160 µm, color-coded).Contrast-reversing gratings were delivered at 2 Hz temporal frequency. The timing of peak cross-correlation remains consistent across bar widths. (B) Mean cross-correlation functions across cells (n = 10 cells), shown for a subset of bar widths (10, 20, 40, and 80 µm). Shaded regions indicate SEM.

Supplementary Table 1. Parameters for the Temporal and Spatial-Temporal Dynamic Synapse Models

Parameter	Description	Temporal Only Model Value	Spatial-Temporal Model Value	Unit
K_{rec}	Vesicle recovery rate	10	10	Hz
K_{rel}	Vesicle release rate	5	5	Hz
b	Release gain	5	5	-
γ	Rectification ratio (exc)	0.3	0.3	-
E_0	Norm. excitation baseline	0.3	0.3	-
α	Presynaptic inhibition Strength [0,1]	0.9	0.9	-
β	Sensitivity of release rate to inhibition	1	1	-
E_0	Baseline excitation	0.3	0.3	-
σ_{exc}	Excitatory subunit center size	-	22	μm
d_{exc}	Excitatory subunit spacing	-	44	μm
$\sigma_{pool,exc}$	Excitatory pooling radius	-	50	μm
σ_{inh}	Inhibitory subunit center size	-	12	μm
d_{inh}	Inhibitory subunit spacing	-	24	μm
$\sigma_{inh,\ surround}$	Inhibitory surround size	-	20	μm
δ	Surround strength onto inhibition subunit	-	0.9	-
$\sigma_{pool,inh}$	Inhibitory pooling radius	-	100	μm
